# Ym1 induces RELMα and rescues IL-4Rα deficiency in lung repair during nematode infection

**DOI:** 10.1371/journal.ppat.1007423

**Published:** 2018-11-30

**Authors:** Tara E. Sutherland, Dominik Rückerl, Nicola Logan, Sheelagh Duncan, Thomas A. Wynn, Judith E. Allen

**Affiliations:** 1 Lydia Becker Institute for Immunology & Infection, Faculty of Biology, Medicine & Health, Manchester Academic Health Science Centre, University of Manchester, Manchester, United Kingdom; 2 School of Biological Sciences, University of Edinburgh, Edinburgh, United Kingdom; 3 Immunopathogenesis Section, Laboratory of Parasitic Diseases, National Institute of Allergy and Infectious Diseases, National Institutes of Health, Bethesda, Maryland, United States of America; 4 Wellcome Centre for Cell-Matrix Research, Faculty of Biology, Medicine & Health, Manchester Academic Health Science Centre, University of Manchester, Manchester, United Kingdom; University of Medicine & Dentistry New Jersey, UNITED STATES

## Abstract

Ym1 and RELMα are established effector molecules closely synonymous with Th2-type inflammation and associated pathology. Here, we show that whilst largely dependent on IL-4Rα signaling during a type 2 response, Ym1 and RELMα also have IL-4Rα-independent expression patterns in the lung. Notably, we found that Ym1 has opposing effects on type 2 immunity during nematode infection depending on whether it is expressed at the time of innate or adaptive responses. During the lung migratory stage of *Nippostrongylus brasiliensis*, Ym1 promoted the subsequent reparative type 2 response but once that response was established, IL-4Rα-dependent Ym1 was important for limiting the magnitude of type 2 cytokine production from both CD4+ T cells and innate lymphoid cells in the lung. Importantly, our study demonstrates that delivery of Ym1 to IL-4Rα deficient animals drives RELMα production and overcomes lung repair deficits in mice deficient in type 2 immunity. Together, Ym1 and RELMα, exhibit time and dose-dependent interactions that determines the outcome of lung repair during nematode infection.

## Author summary

Immune cells produce molecules that limit infections, attract other immune cells or repair tissue damage. Two such molecules, Ym1 and RELMα, are abundantly produced in mice during infection and injury. Related human molecules are strongly associated with many chronic diseases and yet we know very little about their function. *Nippostrongylus brasiliensis* is a parasitic worm that migrates through the lung causing tissue damage, which is accompanied by a strong early inflammatory response. Subsequently the lung is rapidly repaired, a process that requires an immune response that is characterised by the production of large amounts of Ym1 and RELMα. Here we show that during the early lung injury phase of infection, Ym1 helps direct the immune response towards repair but once repair has been initiated, Ym1 acts to limit excessive immune activation. Additionally, Ym1 enhances RELMα production in the lung and together, these molecules contribute to lung repair. Therefore Ym1 and RELMα have distinct regulatory functions depending on the stage of injury or infection, and additionally can act directly to repair injured tissues. These findings demonstrate the diverse functions of Ym1 and RELMα with wider implications toward understanding the relationship between host immune responses and tissue repair.

## Introduction

Type 2 immunity is an important component of host defense against helminth infections [1]. Murine infection with the lung migrating nematode, *Nippostrongylus brasiliensis*, elicits a strongly polarised type 2 response, characterised by IL-4, IL-13, IL-5 and IL-9 cytokines. This response is induced once larvae migrate through the airways to take up residence in the intestine [2]. Binding of IL-4/IL-13 via the IL-4 receptor subunit alpha (IL-4Rα) is essential for the efficient induction of a type 2 response. IL-4Rα-signaling has been shown to be important not only for parasite expulsion [3] but also for restoration of lung tissue integrity following larval migration and acute neutrophilic inflammation [4]. After engagement of IL-4Rα in mice, the expression of chitinase-like protein, Ym1 (*Chil3*) and resistin-like molecule alpha (RELMα; *Retnla*) are upregulated in many cell types, including epithelial cells. Due to their abundant expression in macrophages, Ym1 and RELMα are hallmarks of the alternatively activated or M(IL-4) phenotype [5–8]. Whilst we do not yet fully understand the interactions and downstream targets of these molecules, it is clear that both Ym1 and RELMα are important regulators of type 2 immunity [9–11]. As such there is a great deal of interest surrounding the function of these effector molecules and their relationship to inflammation and pathology.

Chitinase-like proteins (CLPs) are structurally related to chitinases, enzymes that are host-protective through their ability to hydrolyse chitin [12]. Loss-of-function mutations following gene duplication of chitinases, rendered CLPs enzymatically inactive and yet mammalian CLPs appear to be major players during inflammation and pathology [13,14]. CLPs are highly expressed during arthritis [15], cancer [16], fibrosis [17], asthma/allergy [18,19] and helminth infection [20,21], conditions that are often but not exclusively associated with type 2 dominated responses. Along with Ym1, RELMα, a cysteine-rich secreted protein, is strongly upregulated during type 2 responses [21,22] and expression of both proteins is typically indicative of a strongly polarized type 2 response. There are contrasting findings regarding the role of RELMα during pathology, with reports indicating RELMα can promote [23,24] or dampen inflammation [10,11] suggesting these roles are highly context dependent. RELMα and Ym1 are often co-expressed in macrophages [6], epithelial cells [25], dendritic cells [26] and neutrophils [27] and in many scenarios, mutually dependent on IL-4Rα [5] and STAT6 signaling [28,29]. Nonetheless, expression of Ym1 (by macrophages and neutrophils) and RELMα (by granulocytes and epithelial cells) is readily detectable in the lungs in the absence of type 2 inflammation [22,30,31] and Ym1 and RELMα can be expressed independently of one another [32–35].

Herein, we aimed to explore the relationship between Ym1 and RELMα in the lungs, both during homeostasis and *N*. *brasiliensis* infection, with a particular emphasis on the contribution of IL-4Rα signaling. Our results revealed that innate IL-4Rα-independent Ym1 plays a role in initiating an appropriate type 2 response that occurs later during infection. Conversely IL-4Rα-dependent Ym1 limited type 2 immune responses. We additionally found that Ym1 was able to promote epithelial derived RELMα and mediate tissue repair, and that these actions occurred even in the absence of IL-4Rα signaling. RELMα was important for lysyl hydroxylase expression and tissue repair in the lung following infection-induced pathology, consistent with the ability of RELMα to orchestrate collagen cross-linking in the skin [36]. Together these results demonstrate differential roles for Ym1 depending on the stage of nematode infection, with the novel finding that it can directly promote repair and induce the pro-fibrotic protein RELMα.

## Results

### IL-4Rα-dependent and -independent pathways contribute to Ym1 and RELMα expression in the lung

**Fig 1:**
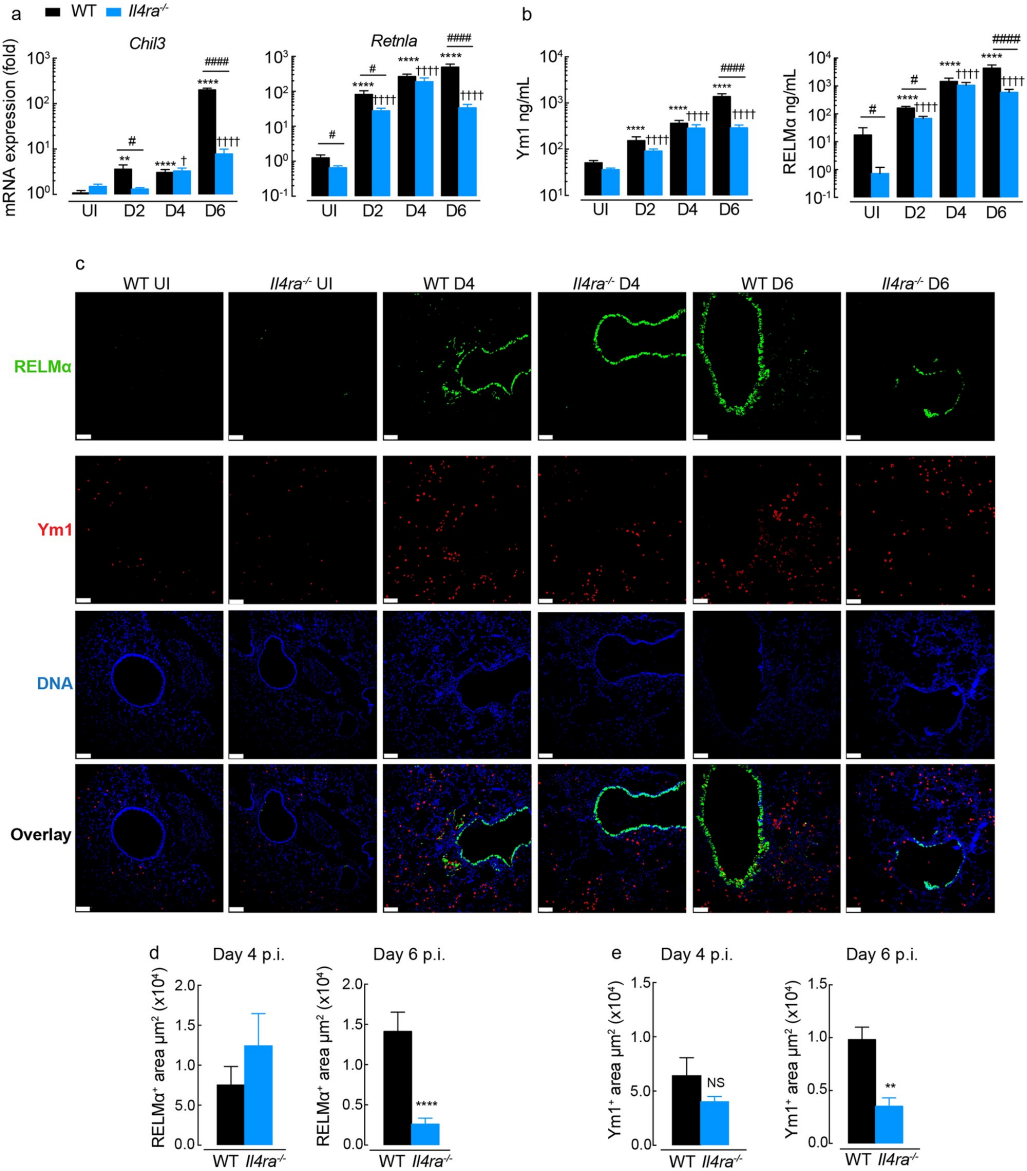
The expression of Ym1 and RELMα in the lungs of mice. **(a)** Amplification of *Chil3* and *Retnla* mRNA in lung tissue from BALB/c WT or *Il4ra*-/- mice left uninfected (UI) or infected with *N*. *brasiliensis* (500 L3’s) and assessed at days 2, 4 and 6 post-infection (results are relative to uninfected WT, set as 1 (10^0^); *n* = 12–6 per group; data are shown as mean ± sem; two-way ANOVA with Tukey multi-comparison test; NS not significant, ****P<0.0001 compared to UI wild-type (WT); ✝✝✝✝ P<0.0001 compared to UI *Il4ra*-/-; #P<0.05 and #### P<0.0001 wild-type compared to *Il4ra*-/- mice; data pooled from 2 independent experiments). **(b)** Ym1 and RELMα levels in the BAL fluid from mice as in **a**. **(c)** Microscopy of lung sections from WT and *Il4ra*-/- BALB/c naive mice or mice infected with *N*. *brasiliensis* at day 4 and 6, stained with the DNA-binding dye (DAPI), blue; Ym1, red; and RELMα, green (scale bars, 70μm; images are representative of n = 6 of 2 independent experiments). (**d**) Quantification of the RELMα^+^ areas in lung sections stained in **c** (*n* = 6 per group; data are shown as mean ± sem; unpaired t test, ****P<0.0001; data representative of 2 independent experiments). (**e**) Quantification of the Ym1^+^ areas in lung sections stained from **c** (*n* = 6 per group; data are shown as mean ± sem; unpaired t test, NS not significant and **P<0.01; data representative of 2 independent experiments).

We examined the IL-4Rα-dependency of Ym1 and RELMα expression in the lung in the naive state and during innate or adaptive type 2 immune responses. Whilst Ym1 and RELMα expression is readily detectable in the uninfected lungs of both wild-type and IL-4Rα-deficient mice (Fig 1a and 1b), *Il4ra*-/- mice have significantly less RELMα compared to wild-type controls (Fig 1a and 1b). Following infection with *N*. *brasiliensis*, the expression and secretion of *Chil3* (Ym1) and *Retnla* (RELMα) in the lung and BAL respectively, increased over time in both *Il4ra*-/- and wild-type mice (Fig 1a and 1b). However, RELMα levels were significantly lower in IL-4Rα-deficient mice at all time points apart from day 4. In contrast to RELMα, there was no significant difference in Ym1 levels between genotypes in naive animals (Fig 1b), but there was an initial delay in upregulation of *Chil3* expression in *Il4ra*-/- at day 2 post-infection (Fig 1a). By day 6 post-infection, a time coinciding with adaptive immunity and an established type 2 response, both RELMα and Ym1 expression was significantly reduced in IL-4Rα-deficient mice compared to BALB/c wild-type mice (Fig 1a and 1b). Nonetheless, for both proteins there were significant increases in expression during infection independent of IL-4Rα signaling.

To determine whether it is specific cell types that maintain expression of Ym1 and RELMα in the absence of IL-4Rα signaling, lung sections were assessed by immunofluorescence (Fig 1c and S1a Fig). Airway epithelial cells are known to make a large contribution to the secreted levels of Ym1 and RELMα during type 2 immune responses in the lungs [10,30]. Consistent with this, RELMα was strongly expressed by lung epithelial cells at day 6 post infection. However, few Ym1^+^ epithelial cells were observed in lung sections and the majority of Ym1 appeared to be expressed in the myeloid compartment (Fig 1c and S1a Fig). RELMα+ myeloid cells could also be identified in lung sections but at a much lower intensity compared to the airway epithelium (Fig 1c and S1a Fig). At day 4, epithelial derived RELMα was largely independent of IL-4Rα expression (Fig 1c and 1d), coinciding with equivalent RELMα protein levels in the BAL of wild-type and *Il4ra*^-/-^ mice (Fig 1b). However, by day 6 post-infection, IL-4Rα-dependence of RELMα expression was evident in the airway epithelium (Fig 1c), and areas of RELMα positivity were significantly reduced in lungs from *Il4ra*^-/-^ compared to wild-type mice (Fig 1d). Similarly, Ym1^+^ staining was reduced in lung sections from *Il4ra*^-/-^ compared to wild-type mice at day 6 (Fig 1e). Intracellular flow cytometry of Ym1 and RELMα was used to determine whether specific myeloid cells were affected by the absence of IL-4Ra signaling (S1b–S1d Fig). In uninfected mice, regardless of IL-4Rα expression, alveolar macrophages and neutrophils made up the predominant pool of Ym1^+^ cells, whilst RELMα expression appeared limited to DC populations and granulocytes (S1c and S1d Fig). Infection led to an unexpected reduction in the frequency of Ym1^+^ alveolar macrophages and neutrophils likely reflective of active secretion of intracellular proteins (S1d Fig). Notably, the loss of Ym1 expression in neutrophils was dependent on IL-4Rα expression suggesting that signaling through the receptor may mediate Ym1 release. Whilst a significant reduction in Ym1+ monocyte-derived dendritic cells (MoDCs) and DCs were observed in IL-4Rα-/- compared to wild-type mice, the overall contribution of these cell types to the pool of secreted Ym1 is likely to be limited (S1d Fig) and probably does not explain the overall reduction in the Ym1+ area in stained lung sections (Fig 1c and 1e). However, routine tissue digestion may not release all myeloid cell populations for flow cytometry with some resident myeloid populations only detectable by staining lung sections. Unlike Ym1, which is predominantly produced by macrophages and neutrophils following infection, many different cell types appear to contribute to RELMα production in the lungs (S1c and S1d Fig). Reduced numbers of RELMα+ interstitial macrophages (IMs), MoDCs, DCs, eosinophils and epithelial cells (S1a, S1c and S1d Fig) were together responsible for reduced RELMα secretion in IL-4Rα-/- mice (Fig 1b).

Together these results demonstrated that high level expression of both Ym1 & RELMα is IL-4Rα-dependent in the context of nematode infection of the lung, extending other studies [25,32,37]. However, they also revealed an important contribution of IL-4Rα-independent pathways for Ym1 and RELMα expression, which was particularly evident for Ym1 prior to full establishment of the adaptive type 2 response. Surprisingly, IL-4Rα-independent expression of RELMα and Ym1 was observed in all cell types examined, with the exception of MoDCs, whereby infection-induced Ym1 was strongly IL-4Rα-dependent.

### Innate versus adaptive Ym1 differentially influences type 2 responses

**Fig 2:**
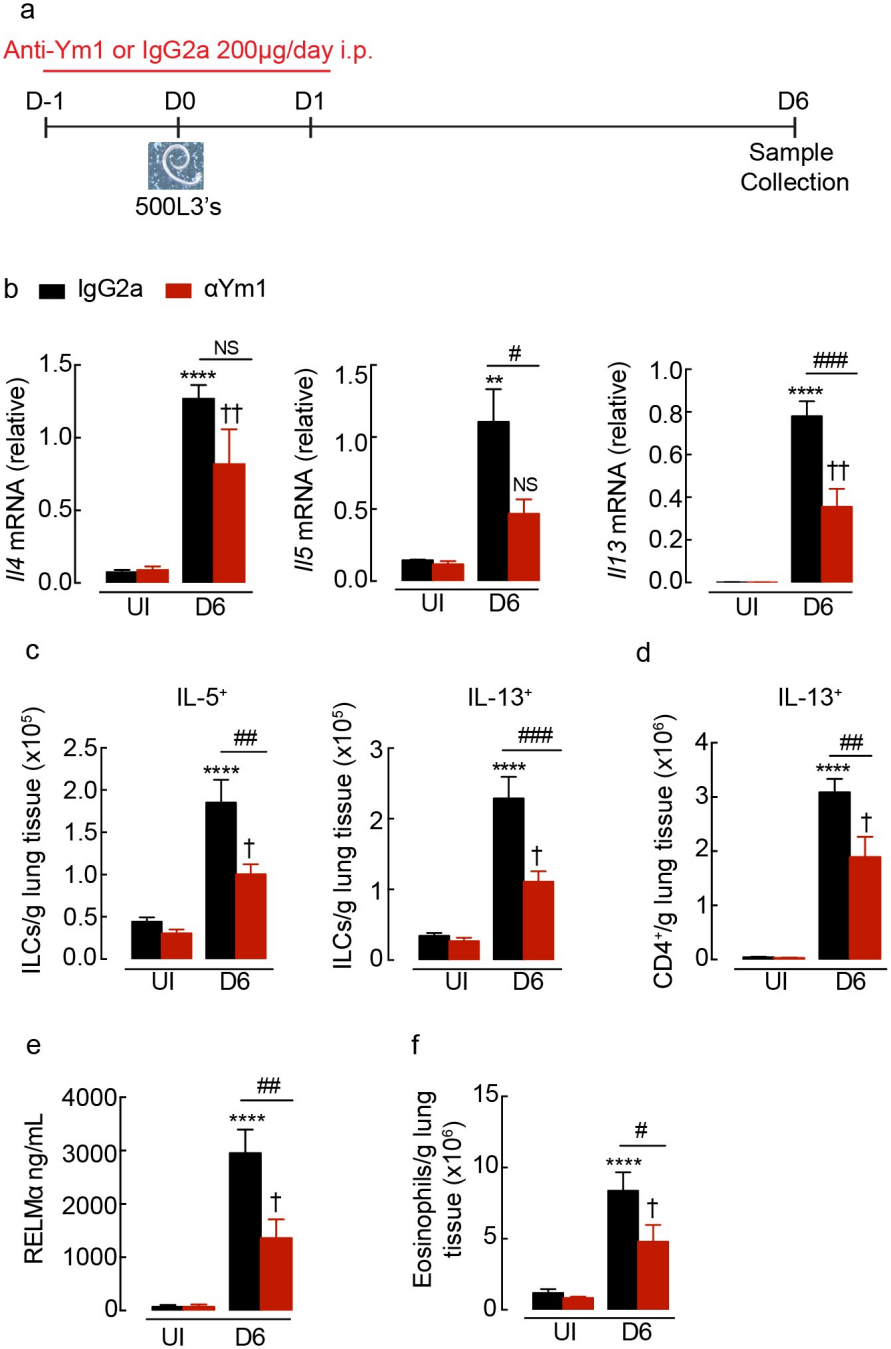
Innate Ym1 promotes type 2 cytokine production in the lung during *N*. *brasiliensis* infection. **(a)** Time-line of infection with *N*. *brasiliensis* (500 L3’s) and dosing with anti-Ym1 or IgG2a. **(b)** Expression of *Il4*, *Il5* and *Il13* mRNA in whole lung tissue of uninfected (UI) or *N*. *brasiliensis* infected mice (D6) treated intraperitoneally with anti-Ym1 or IgG2a (*n* = 6 per group; data are shown as mean ± sem; two-way ANOVA with Sidak multi-comparison test; NS not significant, **P<0.01 ****P<0.0001 compared to UI IgG2a treated; ✝✝ P<0.01 compared to UI anti-Ym1; #P<0.05 and ### P<0.001 IgG2a infected compared to anti-Ym1 infected mice; data representative of 2 independent experiments). **(c)** The number of ILC2s expressing intracellular IL-5 or IL-13 within the lungs of mice as in **b**. Single cell lung suspensions were stimulated ex vivo with PMA and ionomycin, graphs show absolute number of cytokine positive cells per g of lung tissue (*n* = 6 per group; data are shown as mean ± sem; two-way ANOVA with Sidak multi-comparison test; ****P<0.0001 compared to UI IgG2a treated; ✝ P<0.05 compared to UI anti-Ym1; ##P<0.01 and ### P<0.001 IgG2a infected compared to anti-Ym1 infected mice; data representative of 2 independent experiments). **(d)** Expression of IL-13 in CD4^+^ T cells from mice as described in **b**. **(e)** RELMα levels secreted into the BAL of mice as in **b** (*n* = 6 per group; data are shown as mean ± sem; two-way ANOVA with Sidak multi-comparison test; ****P<0.0001 compared to UI IgG2a treated; ✝ P<0.05 compared to UI anti-Ym1; ##P<0.01 IgG2a infected compared to anti-Ym1 infected mice; data representative of 2 independent experiments). **(f)** Absolute number of eosinophils from lungs of mice as in **b** (*n* = 6 per group; data are shown as mean ± sem; two-way ANOVA with Sidak multi-comparison test; ****P<0.0001 compared to UI IgG2a treated; ✝ P<0.05 compared to UI anti-Ym1; #P<0.05 IgG2a infected compared to anti-Ym1 infected mice; data representative of 2 independent experiments).

We have previously found that IL-4Rα-independent Ym1 expression during the steady state and early *N*. *brasiliensis* infection (days 0–4) drives expansion of innate γδ T cell populations expressing IL-17A [9]. In that study we found that increased IL-17A was needed for the induction of a competent type 2 response [9]. We therefore hypothesised that innate Ym1 might regulate the subsequent type 2 response during nematode infection. To test this, *N*. *brasiliensis* infected BALB/c wild-type mice were administered intraperitoneally with a neutralising mouse monoclonal antibody against Ym1 or an isotype matched control antibody (Fig 2a) [9,38]. At day 6 post-infection the increase in *Il5* and *Il13* mRNA expression in total lung was significantly reduced following anti-Ym1 treatment whilst *Il4* was not significantly altered (Fig 2b). As both innate lymphoid cells (ILCs) and Th2 cells are major producers of type 2 cytokines during infection in the lung, we examined these two cell populations following PMA and ionomycin stimulation of single cell suspensions. As expected, the absolute number of ILCs and CD4+ T cells expressing type 2 cytokines were increased in the lungs following infection, with approximately 10-fold greater numbers of CD4^+^ T cells than ILCs (Fig 2c and 2d). Anti-Ym1 significantly reduced the numbers of IL-5- and IL-13-producing ILCs in the lung (Fig 2c). Reduced ILCs together with a significant reduction in the numbers of IL-13^+^ CD4^+^ T cells (Fig 2d), likely contributed to the overall reduction in type 2 cytokine expression in the lung (Fig 2b). The effect of Ym1 on the type 2 response was not restricted to the lungs of infected mice, as anti-Ym1 treatment also reduced basal splenocyte cytokine secretion and anti-CD3 stimulated IL-5 and IL-13 but had no effect on IL-4 secretion (S2a Fig). Consistent with the dependence of RELMα expression on IL-4Rα signaling described above (Fig 1 and S1 Fig), RELMα secretion was reduced following anti-Ym1 treatment (Fig 2e). In addition, eosinophil influx in infection, a response highly dependent on IL-5 [39], was also reduced following anti-Ym1 treatment (Fig 2f).

**Fig 3:**
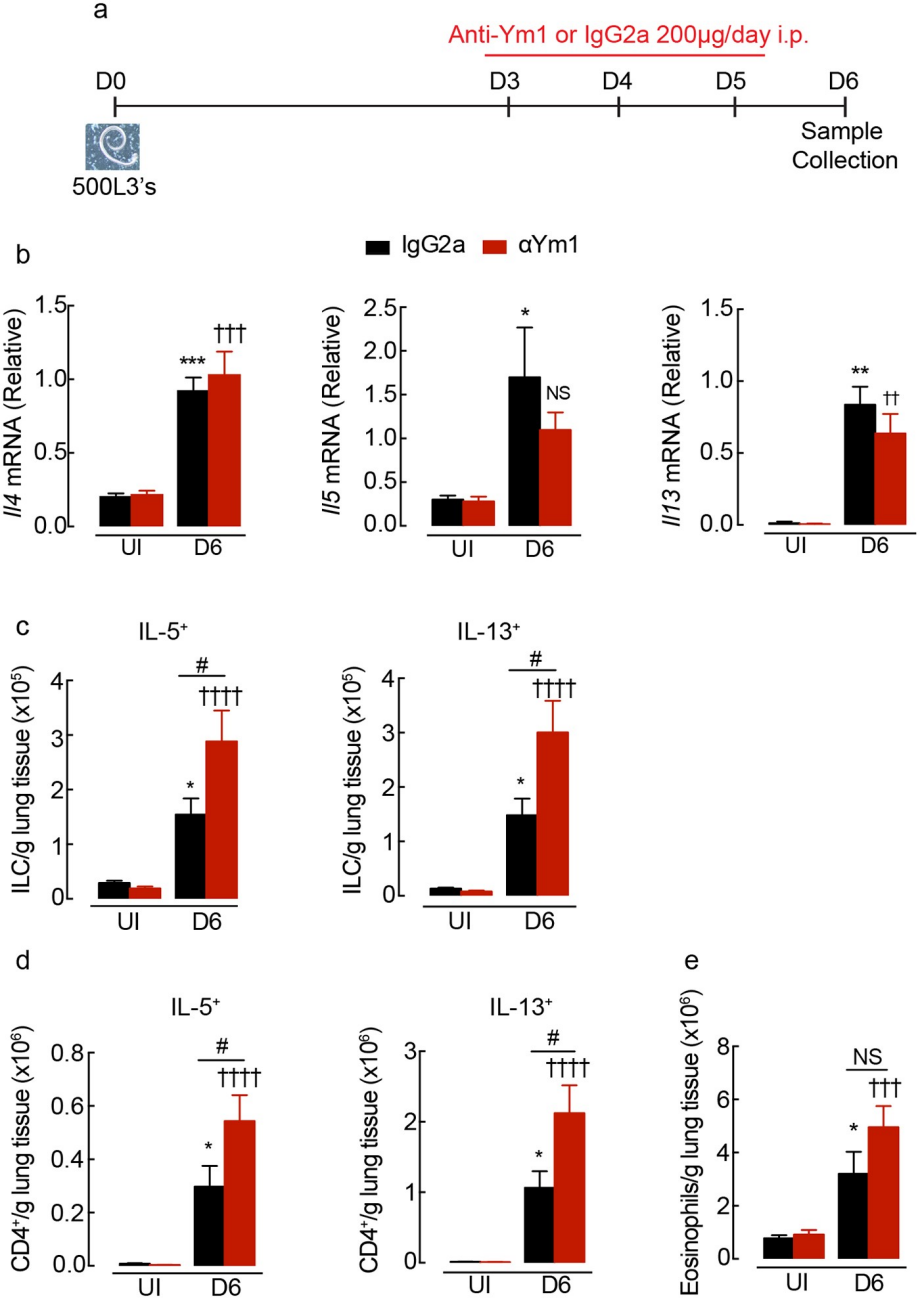
Adaptive Ym1 limits type 2 cytokine production in the lung during infection. **(a)** Time-line of infection with *N*. *brasiliensis* and dosing with anti-Ym1 or IgG2a. **(b)** Expression of *Il4*, *Il5* and *Il13* mRNA in whole lung tissue of uninfected (UI) or *N*. *brasiliensis* (500 L3’s) infected mice (D6) treated intraperitoneally with anti-Ym1 or IgG2a (*n* = 6 per group; data are shown as mean ± sem; two-way ANOVA with Sidak multi-comparison test; NS not significant, *P<0.05, **P<0.01 ***P<0.001 compared to UI IgG2a treated; ✝✝ P<0.01, ✝✝✝P<0.001 compared to UI anti-Ym1; data representative of 3 independent experiments). **(c)** The number of ILCs expressing intracellular IL-5 or IL-13 within the lungs of mice as in **b**. Single cell lung suspensions were stimulated ex vivo with PMA and ionomycin, graphs show absolute number of cytokine positive cells per g of lung tissue (*n* = 6 per group; data are shown as mean ± sem; two-way ANOVA with Sidak multi-comparison test; *P<0.05 compared to UI IgG2a treated; ✝✝✝P<0.001, ✝✝✝✝ P<0.0001 compared to UI anti-Ym1; NS not significant, #P<0.05 IgG2a infected compared to anti-Ym1 infected mice; data representative of 3 independent experiments). **(d)** Expression of IL-5 and IL-13 in CD4^+^ T cells from mice as described in **b** (*n* = 6 per group; data are shown as mean ± sem; two-way ANOVA with Sidak multi-comparison test; *P<0.05 compared to UI IgG2a treated; ✝✝✝✝ P<0.0001 compared to UI anti-Ym1; #P<0.05 IgG2a infected compared to anti-Ym1 infected mice; data representative of 3 independent experiments). **(e)** Absolute number of eosinophils from lungs of mice as in **b** (*n* = 6 per group; data are shown as mean ± sem; two-way ANOVA with Sidak multi-comparison test; *P<0.05 compared to UI IgG2a treated; ✝✝✝ P<0.001 compared to UI anti-Ym1; NS not significant, IgG2a infected compared to anti-Ym1 infected mice; data representative of 3 independent experiments).

Our data showed that innate sources of Ym1 promoted the development of type 2 immunity leading us to examine whether Ym1 also regulated type 2 immunity once the adaptive type 2 response was initiated (> day 4). We therefore administered anti-Ym1 to *N*. *brasiliensis* infected mice between days 3–5 (Fig 3a). Anti-Ym1 treatment at this later stage of infection had no effect on the expression of *Il4*, *Il5* and *Il13* in whole lung tissue (Fig 3b). In uninfected animals, type 2 cytokine production by ILCs contribute to greater than half IL-13 and IL-5 production in the lungs (S2b Fig). However, in contrast to neutralising innate Ym1 (Fig 2), neutralising adaptive Ym1 resulted in a significant increase in the absolute numbers, of IL-5^+^ and IL-13^+^ ILCs and CD4^+^ T cells in the lungs of infected mice (Fig 3c and 3d), but did not change the proportion of cells that contributed to IL-5 and IL-13 production (S2b Fig). The effects of anti-Ym1 treatment were not restricted to the lungs, as IL-4, IL-5 and IL-13 secretion from splenocyte restimulation were also increased in treated mice (S2c Fig). Despite the small but significant increases in type 2 cytokines, the absolute numbers of eosinophils in the lungs were not altered by anti-Ym1 treatment (Fig 3e), nor was there an impact on parasite recovery (S2d Fig). Overall, our results demonstrate that Ym1 regulates type 2 cytokine producing ILCs and Th2 cell numbers in the lungs, but with opposing outcomes depending on the stage of the immune response.

## Ym1 during adaptive immunity is required for lung tissue repair

**Fig 4:**
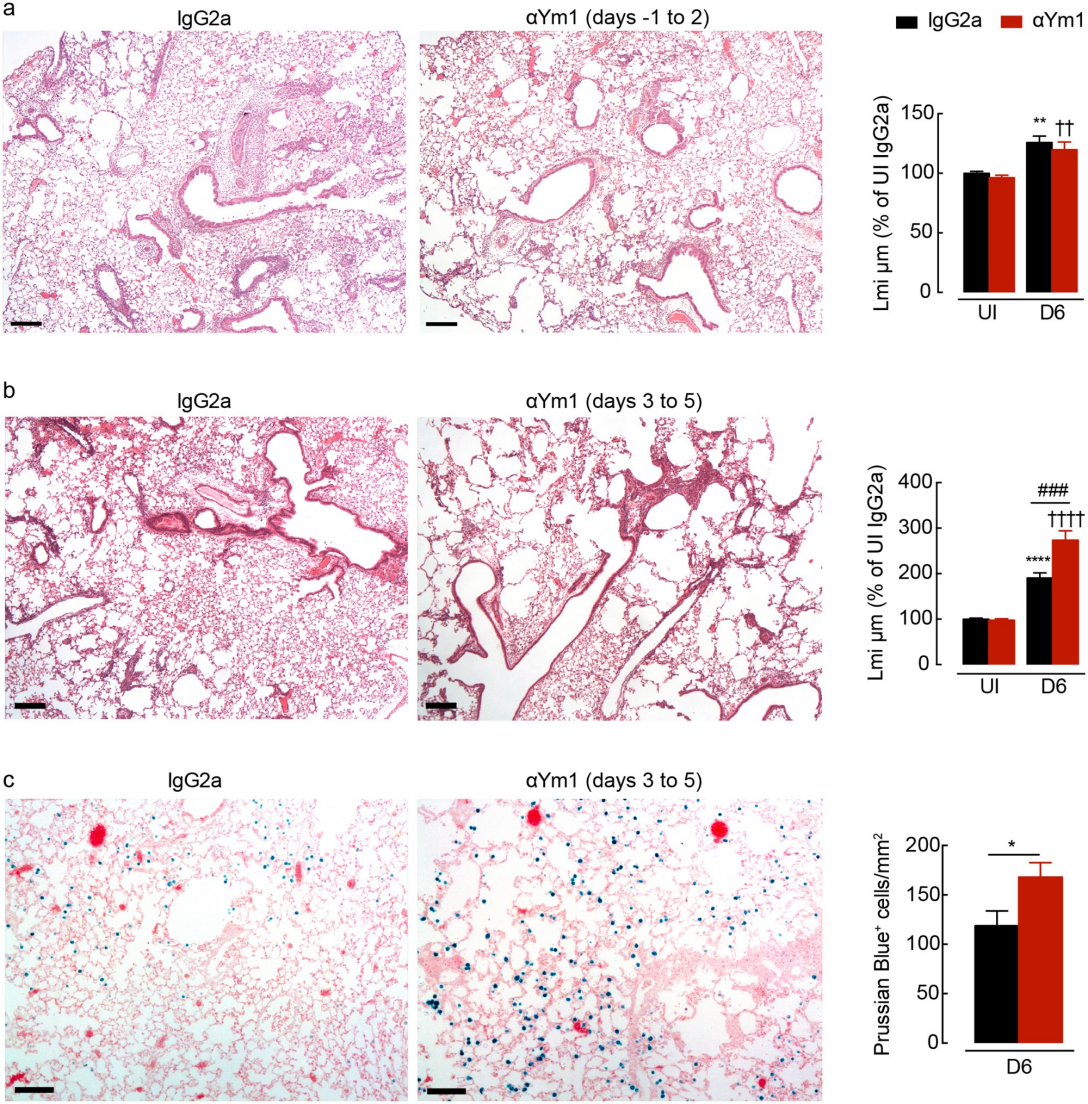
Adaptive Ym1 is required for rapid repair of the lung following helminth infection. **(a)** Microscopy of lung sections from mice uninfected or *N*. *brasiliensis* (500 L3’s) infected mice (day 0) treated intraperitoneally with anti-Ym1 or IgG2a (days -1 to +2) and collected at day 6, stained with hematoxylin and eosin (images are representative of *n* = 6, scale bars, 200μm; graph shows quantification of lung damage as linear means intercept (Lmi), data normalised to average Lmi in uninfected IgG2a treated mice, *n* = 6 per group; data are shown as mean ± sem; two-way ANOVA with Sidak multi-comparison test; **P<0.01 compared to UI IgG2a treated; ✝✝ P<0.01 compared to UI anti-Ym1; data representative of 2 independent experiments). (**b**) Microscopy of lung sections from mice uninfected or *N*. *brasiliensis* infected mice (day 0) treated intraperitoneally with anti-Ym1 or IgG2a (days 3 to 5) and collected at day 6, stained with hematoxylin and eosin (images are representative of *n* = 6, scale bars, 200μm; graph shows quantification of lung damage as linear means intercept (Lmi), data normalised to average Lmi in uninfected IgG2a treated mice, *n* = 6 per group; data are shown as mean ± sem; two-way ANOVA with Sidak multi-comparison test; ****P<0.0001 compared to UI IgG2a treated; ✝✝✝✝ P<0.0001 compared to UI anti-Ym1; ###P<0.001 IgG2a infected compared to anti-Ym1 infected mice; data representative of 3 independent experiments). **(c)** Microscopy of lung sections from mice as in **b**, stained with prussian blue. Graph shows quantification of the number of prussian blue positive cells per area of lung. (images are representative of *n* = 6, scale bars, 100μm, *n* = 6 per group; data are shown as mean ± sem; unpaired t test, *P<0.05; data representative of 3 independent experiments).

IL-4Rα-mediated macrophage responses have been shown to be critical factors for the repair process following *N*. *brasiliensis* larval migration [4]. As blockade of innate Ym1 led to reductions in the type 2 response, we assessed whether innate Ym1 blockade also reduced tissue repair in infected mice. Histological examination of the lungs from control mice 6 days after infection revealed evident peribronchial and perivascular inflammation with minimal areas of alveolar destruction visible (Fig 4a). Alveolar damage, quantified via linear means intercept, revealed an equivalent degree of repair in mice where innate Ym1 was blocked compared to control mice (Fig 4a). Thus despite early anti-Ym1 treatment reducing the type 2 cytokine response (Fig 2b–2d) lung healing proceeded normally, suggesting that even an impaired type 2 response is sufficient for repair.

Ym1 itself has long been thought to play a role in tissue repair supported by the notion that Ym1 binds components of the extracellular matrix [40,41] and is strongly expressed during acute injury [30,34]. Therefore, we examined the lung tissue from infected mice treated with anti-Ym1 during days 3–5 post-infection (Fig 4b). Having established in this setting that anti-Ym1 causes a slight but significant enhancement of the type 2 response (Fig 3), any effects of Ym1 blockade on lung repair would not be due to reduced type 2 cytokines. Histological examination of the lungs from treated mice revealed extensive areas of alveolar damage at day 6 relative to control treated mice (Fig 4b). Quantifying the results via linear means intercept indicated a significantly greater level of injury in anti-Ym1 treated mice, at a time point in which the lungs from IgG2a-treated mice had already undergone extensive repair (Fig 4b). In addition, anti-Ym1 treatment increased the numbers of haemosideran-laden macrophages in the lungs, as indicated by Prussian blue positive cells (Fig 4c), suggesting ongoing vascular damage and capillary microbleeding. Thus, our results provide for the first time, evidence that Ym1 can directly promote tissue repair.

### Ym1 regulates the expression of RELMα

**Fig 5:**
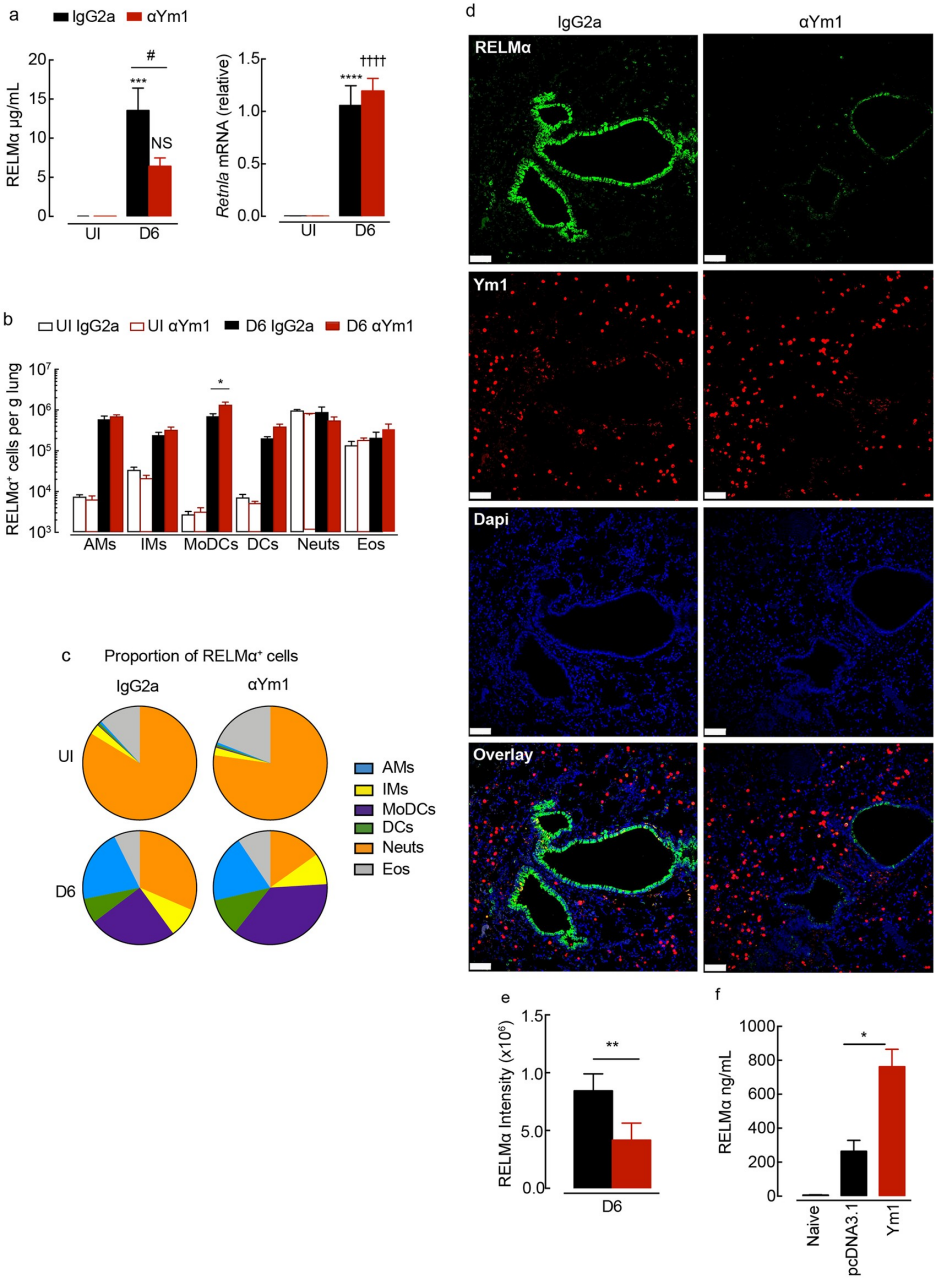
Ym1 stimulates epithelial-derived RELMα during infection with *N*. *brasiliensis*. (**a**) RELMα levels in the BAL fluid or *Retnla* expression in lung tissue from mice uninfected (UI) or *N*. *brasiliensis* (500 L3’s) infected mice (D6) treated intraperitoneally with anti-Ym1 or IgG2a (days +3 to +5) and collected at day 6 (*n* = 6 per group; data are shown as mean ± sem; two-way ANOVA with Sidak multi-comparison test; ***P<0.001, ****P<0.0001 compared to UI IgG2a treated; NS not significant, ✝✝✝✝ P<0.0001 compared to UI anti-Ym1; #P<0.05 IgG2a infected compared to anti-Ym1 infected mice; data representative of 3 independent experiments). (**b**) Total numbers of RELMα+ myeloid cell populations per g of lung tissue from mice as in **a**, analysed by intracellular flow cytometry (*n* = 6 per group; data are shown as mean ± sem; level of RELMα positivity was set from cells stained with rabbit IgG isotype; two-way ANOVA with Sidak multi-comparison test; data representative of 3 independent experiments; AMs, alveolar macrophage; IMs, interstitial macrophage; MoDCs, monocyte derived dendritic cells; DCs, dendritic cells; Neuts, neutrophils; Eos, eosinophils. (**c**) Pie chart showing the proportion of different RELMα^+^ cell populations in lung myeloid cells from **b**. (**d**) Microscopy of lung sections from infected mice as in **a** stained with the DNA-binding dye (DAPI), blue; Ym1, red; and RELMα, green. (Images are representative of 6 individual mice per group; scale bars, 70μm). (**e**) Quantification of the fluorescent intensity of RELMα in lung sections stained from **c** (*n* = 6 per group; data are shown as mean ± sem; unpaired t test, **P<0.01; data representative of 2 independent experiments). **(f)** RELMα levels in the BAL fluid collected at 48hrs from BALB/c mice transfected intranasally with glucose or pcDNA3.1 or Ym1 plasmid (20μg) (*n* = 6–10 per group; data are shown as mean ± sem; one way ANOVA with Sidak multi comparison test, *P<0.05; data were pooled from 2 independent experiments).

Herein, we have observed that Ym1 can regulate type 2 immunity (both positively and negatively) depending on timing. In addition, we found that Ym1 mediated lung repair. RELMα is similarly implicated in both type 2 regulation and tissue repair [36] [10,11]. Ym1 and RELMα are often co-expressed and we have previously observed that RELMα production follows increases in Ym1 [34]. These observations together, led us to consider the possibility that Ym1 may act in part through the induction of RELMα. Strongly supporting this hypothesis was our finding that RELMα levels in the BAL fluid of *N*. *brasiliensis* infected wild-type mice were significantly reduced following anti-Ym1 treatment (day 3–5), an effect not observed on whole lung mRNA expression suggesting post transcriptional regulation (Fig 5a). Importantly, this result cannot be explained by an altered type 2 response, as the timing of anti-Ym1 treatment enhanced IL-5 and IL-13 production (Fig 3), which would be expected to increase RELMα expression. We examined the intracellular expression of RELMα in lung myeloid cells and observed no reduction in RELMα positivity between IgG2a and anti-Ym1 treated infected mice (Fig 5b and 5c). Only a significant increase in the number of RELMα+ MoDCs was seen in the lungs of infected mice following anti-Ym1 treatment. Additionally, anti-Ym1 treatment reduced the proportion of RELMα+ neutrophils (Fig 5c), an effect that likely reflects the reduction in type 2 cytokines as seen in IL-4Rα-/- mice (S1d Fig). In contrast, quantification and visual inspection of RELMα expression by the airway epithelium in histological sections showed that neutralising Ym1 significantly reduced RELMα+ fluorescent intensity (Fig 5d and 5e). Thereby, our data demonstrated an ability of Ym1 to enhance RELMα production, particularly from epithelial cells, independent of altered type 2 cytokine expression. Of note, the enhanced type 2 response itself, may be explained by diminished RELMα in anti-Ym1 treated mice, as RELMα has been shown to suppress Th2 cells [10,11].

We next tested whether Ym1 alone was sufficient to induce RELMα using an in vivo transfection approach. Wild-type BALB/c mice were intranasally administered a plasmid encoding Ym1, which led to a specific upregulation of *Chil3* mRNA expression in BAL cells relative to pcDNA3.1 transfected control mice [9]. Over-expression of Ym1 into the lungs of wild-type mice resulted in a significant increase in RELMα protein secreted into the BAL fluid 48 hrs post-transfection (Fig 5f) suggesting Ym1 expression alone was sufficient to regulate RELMα levels.

### Ym1 induces RELMα and aids tissue repair independently of IL-4Rα signaling

**Fig 6:**
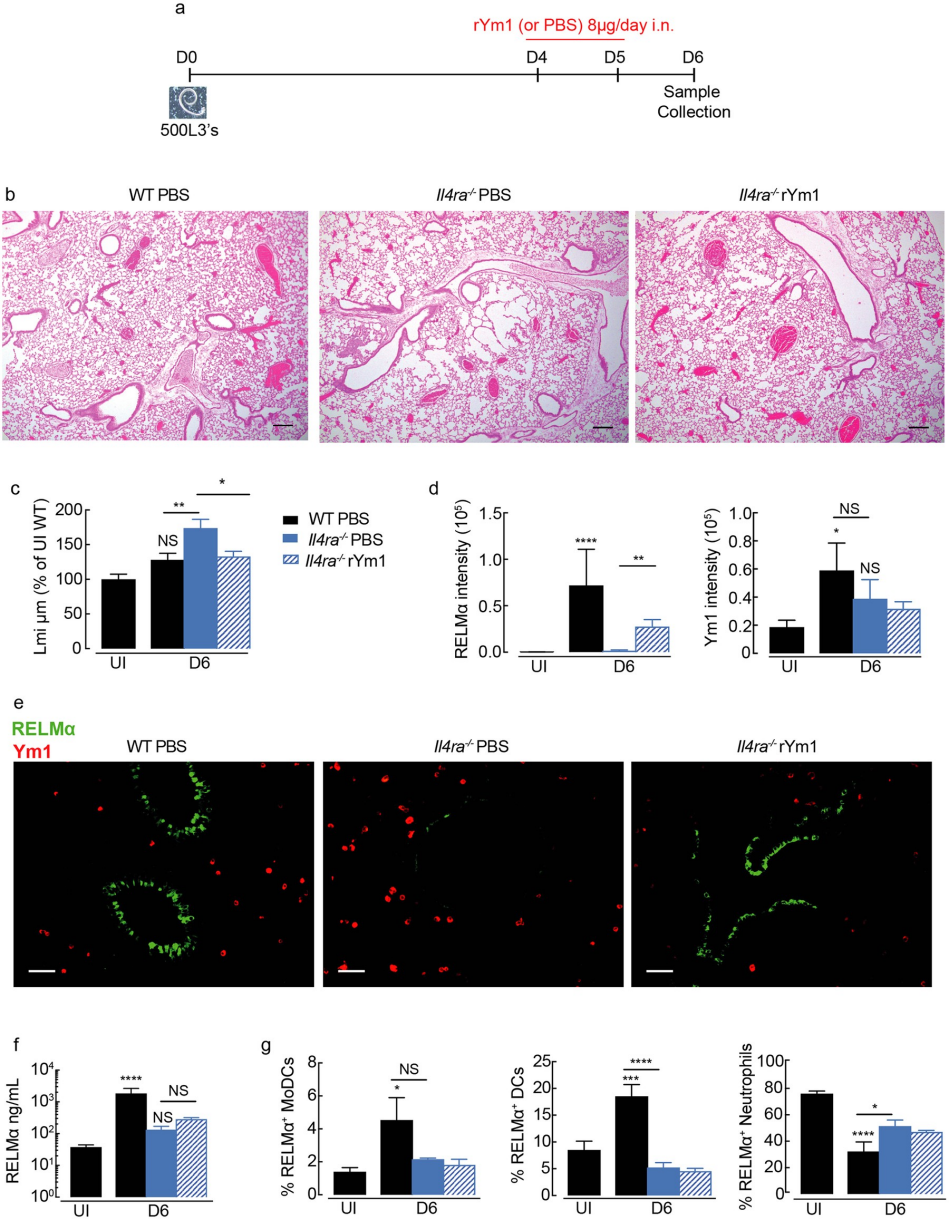
Ym1 regulates tissue repair and RELMα independently of IL-4Rα. **(a)** Time-line of infection with *N*. *brasiliensis* and dosing with rYm1 (8μg) or PBS. (**b**) Microscopy of lung sections from *N*. *brasiliensis* infected (250L3, s.c.) wild-type C57BL/6 or IL-4Rα^-/-^ C57BL/6 mice (day 0) treated intranasally with recombinant Ym1 (8μg) or PBS (days 4 and 5) at day 6 post-infection, and stained with hematoxylin and eosin (images are representative of *n* = 5–6, scale bars, 200μm. (**c**) Quantification of lung damage as linear means intercept (Lmi), data normalised to average Lmi in uninfected wild-type PBS treated mice as in **b**, *n* = 6 per group; data are shown as mean ± sem; one-way ANOVA with Sidak multi-comparison test; NS not significant, *P<0.05 and **P<0.01 compared to UI PBS treated mice. (**d**) Quantification of the fluorescent intensity of RELMα and Ym1 in lung sections in e stained from mice as in **b** (*n* = 6 per group; data are shown as mean ± sem; one-way ANOVA with Sidak multi-comparison test, NS not significant, *P<0.05, **P<0.01 and ****P<0.0001). (**e**) Microscopy of lung sections from infected mice as in **b** stained with Ym1, red; and RELMα, green. (Images are representative of 5–6 individual mice per group; fluorescent intensity quantified in d; scale bars, 50μm). (**f**) RELMα levels in the BAL fluid collected from mice in **b** (*n* = 5–6 per group; data are shown as mean ± sem; one way ANOVA with Sidak multi comparison test, NS not significant, *P<0.05 and ****P<0.00001). (**g**) Frequency of RELMα+ myeloid cells in lung tissue from mice as in **b**, analysed by intracellular flow cytometry (*n* = 6 per group; data are shown as mean ± sem; level of RELMα positivity was set from cells stained with rabbit IgG isotype; MoDCs, monocyte-derived dendritic cells; DCs, dendritic cells.

Type 2 responses are essential for rapid resolution of tissue pathology and as such, lungs from mice deficient in IL-4Rα signaling exhibit a profound failure to repair following *N*. *brasiliensis* infection [4]. However, changes to type 2 cytokine responses following anti-Ym1 treatment could not explain reduced RELMα and delayed tissue repair, although the altered immune response may be a consequent of enhanced tissue damage. We therefore asked whether Ym1 could enhance tissue repair and/or alter RELMα expression independently of the type 2 response. Physiologically relevant levels of recombinant Ym1 observed in the BAL during *N*. *brasiliensis* infection (S2e Fig) and lung inflammation [42], was delivered to IL-4Rα-deficient animals at the time when repair in wild-type mice would usually occur (days 4 and 5) and responses were examined at day 6 post-infection (Fig 6a). As expected, IL-4Rα-/- mice showed enhanced tissue damage, coinciding with a failure to repair the lungs following infection (Fig 6b and 6c). Remarkably, intranasal administration of Ym1 alone was enough to reverse the effects of loss of IL-4Rα and enhance tissue repair to the levels seen in wild-type mice (Fig 6b and 6c). Importantly, accelerated lung repair in Ym1 treated mice did not reflect altered worm burdens in *Il4ra*-/- mice (S2f Fig). Furthermore, Ym1 specifically increased expression of epithelial cell derived RELMα independently of the IL-4Rα (Fig 6d and 6e). Total RELMα secretion into the BAL was not significantly increased in IL-4Rα^-/-^ mice treated with Ym1 (Fig 6f) despite changes to epithelial RELMα production (Fig 6d and 6e). However, this likely reflects the inability of Ym1 to induce RELMα in myeloid cell populations (Fig 6g). Together, this data confirms the ability of Ym1 to aid tissue repair and stimulate epithelial-derived RELMα independently of IL-4Rα signaling and type 2 cytokines.

### RELMα is important for controlling tissue repair rate with an unexpected role for RELMα dose

**Fig 7:**
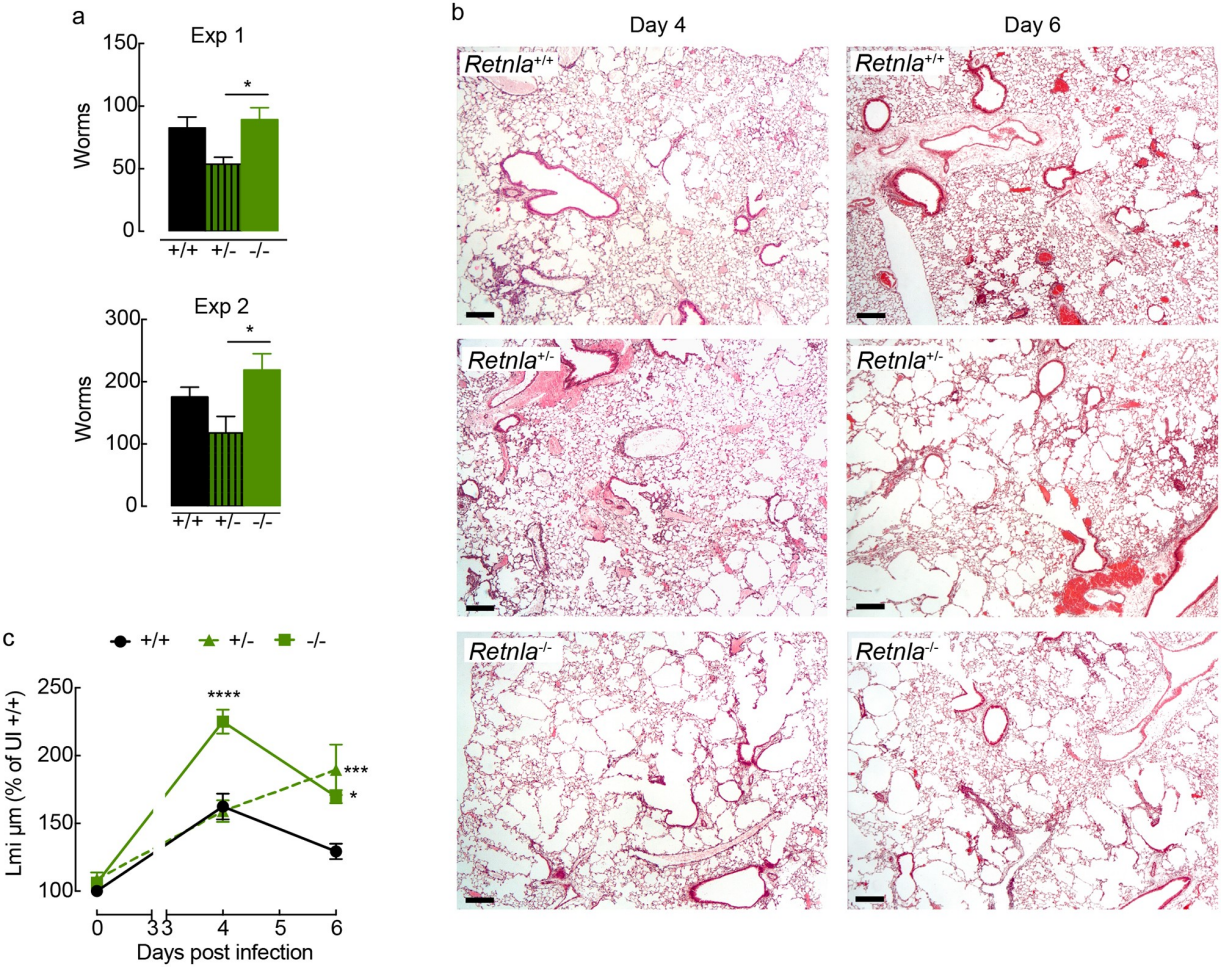
RELMα is required for rapid repair of the lungs following infection with *N*. *brasiliensis*. **(a)** The numbers of worms in the small intestine of littermate control +/+, +/- and -/- *Retnla* mice infected with *N*. *brasiliensis* (500 L3’s) counted at day 4 post-infection (*n* = 6–8 per group; data are shown as mean ± sem; one way ANOVA with Sidak multi comparison test, *P<0.05). (**b**) Microscopy of lung sections from littermate control *Retnla* mice uninfected or infected with *N*. *brasiliensis* collected at day 4 or day 6 post-infection, and stained with hematoxylin and eosin. (images are representative of *n* = 6–8 and 2 independent experiments, scale bars, 200μm) (**c**) Quantification of lung damage, calculated as linear means intercept and values normalised to Lmi in uninfected +/+ mice (*n* = 6–21 per group; data are shown as mean ± sem; two-way ANOVA with Sidak multi-comparison test; *P<0.05 and ***P<0.001 compared to *Retnla* +/+ infected mice; data are pooled from 2 independent experiments).

RELMα is known to be an important player in skin repair [36], and we find here that Ym1 promotes lung repair (Figs 4 and 6) while inducing RELMα production by epithelial cells (Figs 5 and 6). It was therefore important to establish the contribution of RELMα to lung repair following *N*. *brasiliensis* infection. Early intestinal worm burdens in *Retnla*^-/-^ versus C57Bl/6 wild-type mice were examined first to ensure the results were not biased by altered numbers of parasites passing through the lungs. At day 4, we expected similar worm burdens in *Retnla*^-/-^ compared to wild-type mice [10] and indeed this was the case (Fig 7a). However, when using heterozygous littermate controls, we unexpectedly found significantly fewer parasite numbers, suggesting that the amount of RELMα differentially impacts on parasite burden. Notably, we routinely detect a large variation in RELMα protein levels in the serum of both naive wild-type and heterozygote mice with up to ~20-fold difference between mice of the same genotype. (S3a Fig). Because variation in the host RELMα status prior to parasite exposure may influence infection outcome we included heterozygotes in all our subsequent analysis of repair.

We examined infected littermate *Retnla* deficient, heterozygous and sufficient mice during the initiation of repair (day 4) after acute lung injury [9], and at a time when IL-4Rα-signaling is thought to be critical for appropriate repair (day 6) [4]. Whilst histological examination of lungs from *Retnla* +/+ and +/- mice showed small areas of damage at day 4 post-infection (Fig 7b), repair of the lung architecture had been initiated following larval passage. Strikingly, there was extensive alveolar deterioration throughout the lung tissue of *Retnla* -/- mice, an effect quantitatively measurable by changes in linear mean intercept (Fig 7c). As infection progressed to day 6, the lung tissue underwent repair in wild-type mice as well as *Retnla* -/- mice, however, the lungs from *Retnla* -/- mice remained visibly more damaged (Fig 7b and 7c). In contrast, the lungs from *Retnla* +/- mice appeared structurally similar to infected wild-type mice at day 4 (Fig 7b and 7c), but failed to maintain the process of repair through day 6 and instead further deteriorated (Fig 7c). Notably, by day 10 post-infection, the lungs of *Retnla* +/- mice had not deteriorated further, but unlike lungs from wild-type mice exhibited only limited signs of repair (S3b and S3c Fig). This failure of *Retnla* +/- to repair their lungs was associated with an overall reduced RELMα expression but did not appear to be associated with restricted expression in a particular cell type, such as the epithelium (S4 Fig). Although Ym1 promoted tissue repair alongside epithelial-derived RELMα, the experiments in heterozygote mice do not provide evidence for a specific RELMα-expressing cell type involved in tissue repair. Rather it appears that RELMα quantity has a significant role in the dynamics of repair, and one possibility is that Ym1 is an important regulator of RELMα protein availability.

### RELMα regulates expression of lysyl hydroxylase in the lung

**Fig 8:**
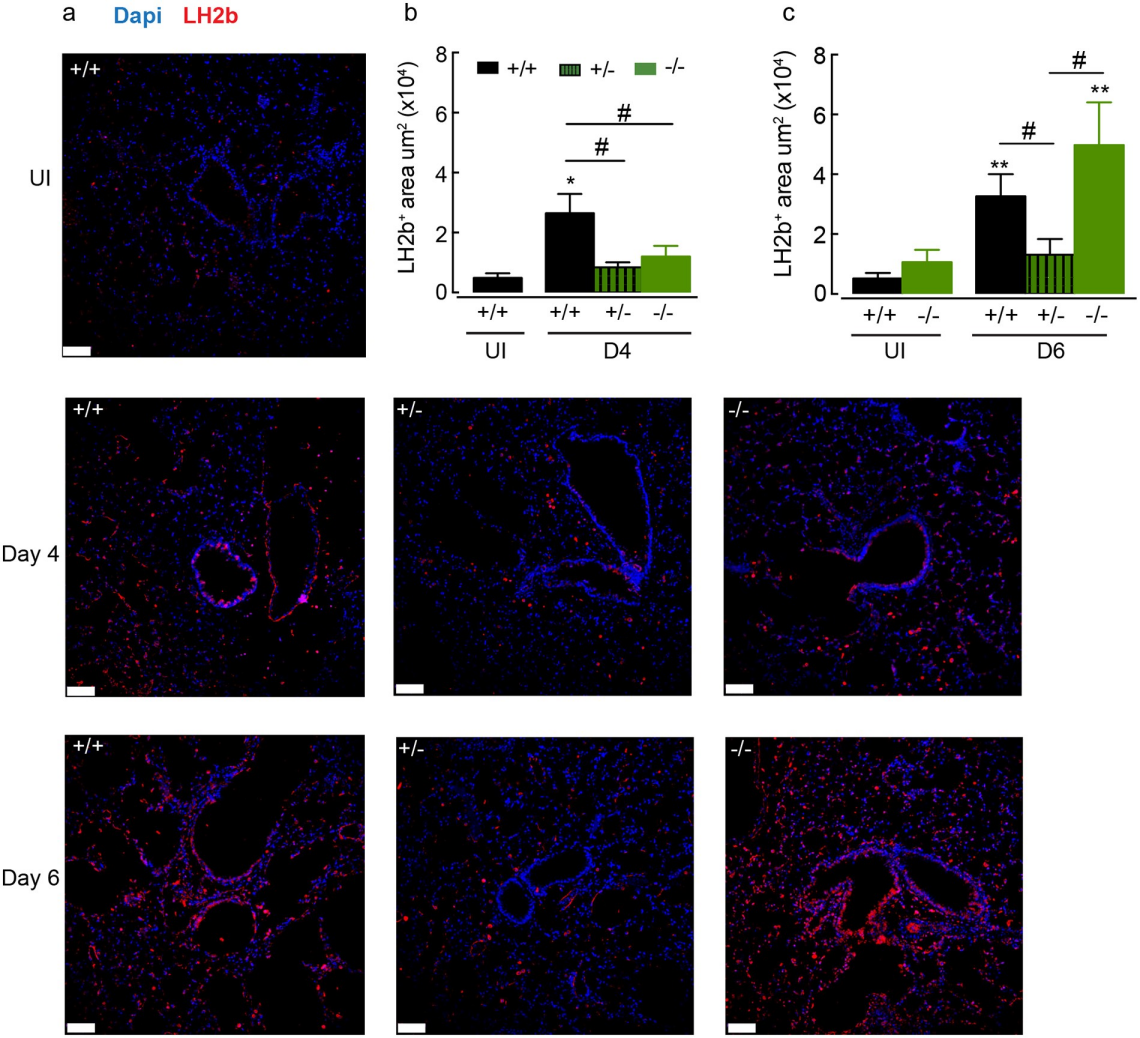
RELMα regulates expression of lysyl hydroxylase 2b during lung repair. **(a)** Microscopy of lung sections from WT and *Retnla* littermate naive mice or mice infected with *N*. *brasiliensis* (500 L3’s; day 4 and day 6), stained with the DNA-binding dye (DAPI), blue and lysyl hydroxylase 2b (LH2b), red. (images are representative of *n* = 5–9 mice per group, scale bars, 70μm). Quantification of positive stained Lh2b area of **(b)** day 4 or **(c)** day 6 infected mice as in **a** (*n* = 5–9 per group; data are shown as mean ± sem; two-way ANOVA with Sidak multi-comparison test; *P<0.05 and **P<0.01 compared to UI *Retnla*+/+ mice; #P<0.05 compared to *Retnla* +/+ infected mice; data are representative from 2 independent experiments).

The ability of RELMα to promote pro-fibrotic collagen cross-linking through increased expression of lysyl hydroxylase has been identified as an important pathway in the generation of an effective wound healing response in the skin [36]. Therefore, we examined the levels of lysyl hydroxylase in the lungs of mice following infection-induced injury in relation to *Retnla* expression. Expression of lysyl hydroxylase 2b (Lh2b) in the lungs of *N*. *brasiliensis* infected wild-type mice at day 4 and day 6 time points was increased relative to uninfected controls (Fig 8) coinciding with tissue repair (Fig 7). Quantification of the area of Lh2b staining revealed a significant reduction in the expression of Lh2b in *Retnla* +/- and -/- mice at day 4 compared to infected wild-type (+/+) mice (Fig 8b). By day 6, when the lungs of RELMα-deficient animals were undergoing repair (Fig 7c), the level of Lh2b expression was equalised to that of a wild-type mouse (Fig 8c). However, Lh2b levels remained low in *Retnla* +/-, reflecting a change in the rate of repair in these mice (Figs 8c and 7c). These results show that RELMα regulates Lh2b expression in the lungs as well as the skin and may play an important role in lung repair by regulating collagen cross-linking following mechanical injury and innate inflammatory insult. However, it appears that the amount of RELMα is an essential factor to sustain repair.

Finally, because we had unexpected results regarding heterozygote mice, we felt it important to re-evaluate in our system, the reports that RELMα negatively regulates Th2 immunity [10,11]. We therefore examined whether type 2 cytokine expression was altered in the lungs of *Retnla* -/- and *Retnla* +/- mice compared to wild-type controls. Although infection led to increases in the numbers of IL-5 and IL-13 producing cells at day 4 and 6, there were no significant differences between *Retnla* genotypes (S5a–S5c Fig). Assessment of IL-4, IL-5 and IL-13 secreted from splenocyte cultures also showed no significant differences between *Retnla* genotypes at day 4 but by day 6 enhanced IL-4, IL-5 and IL-13 was detected in *Retnla* -/- compared to wild type mice and *Retnla* +/- (S6a–S6c Fig). Furthermore, when CD4+ T cell type 2 responses were measured in the lungs at day 10 post-infection, *Retnla* +/- mice exhibited significantly increased numbers of IL-4+ and IL-13+ CD4+ T cells (S5d Fig). These results support the finding that RELMα can negatively regulate the adaptive type 2 response [10,11], but the effect appears to be dependant on the time of infection and is perhaps reflective of an immune response to control ongoing tissue damage in *Retnla* +/- mice.

## Discussion

CLPs are intriguing molecules at the forefront of Th2-type immunopathology, yet their biological functions remain mostly conjectural. We show here that Ym1 produced in the lung during the adaptive response to *N*. *brasiliensis* infection facilitates rapid tissue repair in a process that does not require IL-4Rα. We also reveal that Ym1 regulates the type 2 immune response in opposite directions depending on whether it is expressed during innate versus adaptive phases. Early in infection, levels of Ym1 were independent of IL-4Rα-signaling. During this phase, Ym1 induces an IL-17A/neutrophilic response but also promotes the development of subsequent type 2 immunity [9]. This finding is consistent with increasing evidence that IL-17A is needed for many type 2 responses [9,43,44]. In contrast, once the adaptive Th2 response was established, IL-4Rα-signaling vastly increased Ym1 production. In this context, Ym1 now limited type 2 responses and reduced IL-5 and IL-13 expression (S7 Fig). This suggests that in addition to acting directly as a repair molecule Ym1 may be an endogenous regulator of the Th2-type balance, important for avoiding allergic disease or fibrosis, the consequences of an overzealous response [45].

The concept that Ym1, and CLPs in general, are involved with wound healing and tissue remodeling is not new [30,41,46] but direct experimental evidence has been lacking. The pro-repair actions of Ym1 likely relate to its ability to bind extracellular matrix (ECM) components such as heparin/heparan sulfate proteoglycans [40,41] and regulate the availability of reparative proteins [47,48]. Consistent with this concept, blockade of Ym1 during the adaptive stage of infection prevented efficient lung repair and delivery of Ym1 to IL-4Rα-deficient mice rescued their failure to rapidly repair. Both these experiments revealed Ym1 as an unexpected driver of epithelial-derived RELMα. RELMα can directly restore skin tissue integrity following sterile wounding [36] and has been implicated in extracellular remodeling [49–51]. It is therefore reasonable to hypothesise that RELMα may be at least in part responsible for the pro-repair effects of Ym1. Consistent with its role in the skin [36] we found RELMα to be a critical regulator of the collagen cross-linking enzyme lysl hydroxylase 2 (LH2b, *Plod2*) in the lungs. Notably, the amount of LH2b protein correlated with the degree of lung repair. LH2b is critical for vascular integrity [36], potentially explaining the microbleeding observed in anti-Ym1 treated animals. Crosslinked collagen mediated by LH2b is more stable and resistant to collagenase cleavage resulting in stiffer tissue structure [52]. Increased *Plod2* mRNA expression has been reported during fibrotic conditions [53,54] implicating *Plod2* as a driver of excessive extracellular matrix remodeling [55,56]. Our work here and in the skin [36], suggest it may also be important for rapid tissue regeneration and repair following injury. Whilst we have not directly explored whether Ym1 influences collagen biosynthesis or degradation, our data suggests Ym1 may regulate collagen fibril formation by controlling the quantity of epithelia-derived RELMα. Interestingly YKL-40, a CLP expressed in humans that has strong functional similarities to murine Ym1, not only contains binding motifs for heparin and hyaluronan [57], major constituents of the extracellular matrix, but also for type I collagen [58]. Moreover, binding of YKL-40 to collagen was shown to alter collagen structure or behaviour in a way that prevented cleavage of fibrils and hence aided collagen stability [58,59]. Thus, CLPs in both humans and mice, may play an important regulatory role in collagen formation and turnover either through direct mechanisms or by regulating factors such as RELMα.

In addition to its effects on *Plod2*, RELMα can both promote IL-17 [23] and suppress Th2 cytokines [10,11] and thus downstream actions of RELMα may also contribute to the immune regulatory properties we observed for Ym1. Inconsistent with this hypothesis, at day 6 post-infection, we did not observe any significant changes to IL-5 and IL-13 mRNA or protein producing CD4 T cells or ILCs in the lungs of infected RELMα-deficient mice during this peak reparative phase. However, at day 10 post-infection our studies using heterozygote mice do support the described negative regulatory function for RELMα. These data are in line with numerous published models of inflammation and remodeling, where the ability of RELMα to negatively regulate type 2 cytokines was clearly evident in some studies [10,11] or not detectable in others [60,61]. Unfortunately, unlike the use of the anti-Ym1 antibody, the RELMα deficient mice do not allow us to separate potentially disparate effects of RELMα on the early vs late type 2 response, which may account for the variable outcomes on type 2 inflammation reported in the literature [10,11,60,61]. Whilst differences in published results may be explained by timing or the model and tissue involved, our studies using deficient, heterozygote and wild-type littermate mice reveal that the amount of RELMα may be a critical determinant of its function. For example, our parasite recovery data suggested that high or low levels of RELMα were less effective for host resistance than intermediate levels. A search of published data reveals large variations in reported serum levels of RELMα during the steady state, from as little as ~2ng/mL to upward of ~500ng/mL [23,24,62,63]. We observed an average of 200ng/mL RELMα in serum of wild-type mice, but there was enormous variation in protein levels that could not be accounted for by sex, age or cage allocation. It would be interesting in future studies to determine whether Ym1 mediates its effects at least partly through regulating RELMα availability, whose functions may rely on critical quantitative thresholds.

The expression of RELMα and Ym1 or related family members during many disease pathologies points toward the breadth of their functions that we are only just starting to uncover. In this study of helminth infection, we have illustrated that one molecule, Ym1, can perform distinct and even opposing functions at different stages of infection. The data also suggest that RELMα may function differently depending on the stage of infection. For example, because RELMα can suppress Th2 cytokines and Th2-driven pathology mediated pathology [10,11] it would be logical to hypothesise that RELMα-deficient mice would exhibit accelerated or enhanced lung repair following *N*. *brasiliensis* infection. However, during the period where a heightened repair response is evident, we observed the opposite, consistent with the reported ability of RELMα to mediate collagen turnover [36] and protect against damaging acute lung inflammation [62]. Many of these apparent contradictions may lie with the distinct function of Ym1 and/or RELMα during innate and adaptive stages of an immune response. However, it remains to be seen how tightly linked Ym1’s functions are to its ability to induce RELMα. In addition, recent data suggest that macrophage-derived RELMα is critical for its regulatory function [64] and we have yet to establish whether the ability of Ym1 to induce RELMα is restricted to epithelial cells. Finally, the changes in Ym1 function over time may largely relate to its ability to bind ECM, the properties of which will change over the course of an immune response. Thus, Ym1 interactions within the ECM may enable context-specific biological functions. The details of Ym1-ECM collaboration in vivo remain unexplored and will be an exciting future challenge.

## Methods

### Ethics statement

All animal experiments were performed in accordance with the UK Animals (Scientific Procedures) Act of 1986 under a Project License (70/8548) granted by the UK Home Office and approved by the University of Manchester Animal Welfare and Ethical Review Body. Euthanasia was performed by carbon dioxide exposure.

### Mice

Wild-type (BALB/c or C57BL/6) mice, *Il4ra* -/- (BALB/c and C57BL/6) mice [65] and *Retnla +/+*, *Retnla +/-* or *Retnla -/-* (C57BL/6) mice [10] were bred at the University of Edinburgh or the University of Manchester. All mice were 7–14 weeks old at the start of the experiment and were housed in individually ventilated cages during experimental procedures. For experiments using *Retnla +/+*, *Retnla +/-* or *Retnla -/-*, mice were bred as littermates and were randomized in cages with investigators blind to mouse identity during necropsy. All experiments used female mice except *Retnla* littermate experiments (Figs 7 & 8 and S3–S6 Figs), which used both sexes.

### Anti-Ym1

Anti-Ym1 and IgG2a isotype matched control antibodies were purified from hybridoma cell lines as described previously [9]. Briefly, anti-Ym1 mouse hybridoma cell line (clone 4D10) was generated by immunising mice with a Ym1 peptide (IPRLLLTSTGAGIID) shown to be neutralizing [66]. The hybridoma cell line (clone 2D12) from European Collection of Cell Cultures was used as the IgG2a isotype-matched control antibody. Antibodies were purified by protein G affinity chromatography using an Akta Prime Plus (GE Healthcare).

### *N*. *brasiliensis* infection

*N*. *brasiliensis* was maintained by serial passage through Sprague-Dawley rats, as previously described [2]. Third-stage larvae (L3) were washed ten times with sterile PBS prior to subcutaneous infection of 500 L3’s or 250 L3’s per mouse (figure legend details *N*. *brasiliensis* infection dose in each experiment). In some experiments, mice were treated intraperitoneally with 200μg anti-Ym1 or IgG2a isotype on days indicated (Figs 2a and 3a). Additional some mice were treated intranasally with recombinant Ym1 (R&D Systems) or PBS on days indicated (Fig 6a). On days 4, 6 and 10 post-infection BAL was performed with 0.25% BSA containing PBS and lungs were taken for further assays and analysis. Single-cell suspensions of splenocytes were stimulated ex vivo with *N*. *brasiliensis* excretory secretory product antigen (1μg/mL or anti-CD3 1μg/mL) for 72 hrs. Cell supernatants were collected and stored at -20°C until further analysis. At day 4 and 6 post-infection, the small intestine was removed from mice and stored in Dulbeccos’ PBS. Intestines were then cut longitudinally along the entire length of the gut and parasite numbers counted manually with the aid of a dissecting microscope.

### In vivo transfection

Wild-type BALB/c mice were administered 20μg pcDNA3.1 (Control) or Ym1 plasmid complexed with in vivo JetPEI (Source Bioscience) intranasally as described previously [9]. BAL fluid was harvested 48 h after transfection. Mice that were not transfected were excluded from the analysis.

### Extraction of RNA and qRT-PCR

A section of the right lung lobe was stored in RNAlater prior to homogenization in Qiazol reagent, or BAL cell pellets were resuspended in Qiazol reagent at the time of necropsy and stored at -80°C until further analysis. RNA was prepared according to manufacturers instructions. Reverse transcription of 0.5μg of total RNA was performed using Tetro reverse transcriptase (Bioline) [9]. Transcripts of genes of interest were measured by qRT-PCR with the Lightcycler 480 II system and Brilliant III SYBR Master mix (Agilent) and specific primer pairs (Table 1). PCR amplification was analysed by the second-derivative maximum algorithm (LightCycler 480 Sw 1.5; Roche) and expression of the gene of interest normalized to expression of housekeeping genes *Rpl13a* or *18srRNA*.

**Table 1:** Primer sequences.

Gene	Forward Primer	Reverse Primer
*Il4* (interleukin 4)	TGCCTGGATTCATCGATAAGCTGCAA	ACGAGTAATCCATTTGCATGATGCTCT
*Il5* (interleukin 5)	ACATTGACCGCCAAAAAGAG	CACCATGGAGCAGCTCAG
*Il13* (interleukin 13)	CCTCTGACCCTTAAGGAGCTTAT	CGTTGCACAGGGGAGTCT
*Retnla* (Resistin-like molecule alpha)	TATGAACAGATGGGCCTCCT	GGCAGTTGCAAGTATCTCCAC
*Chil3* (Chitinase-like protein 3)	ACCTGCCCCGTTCAGTGCCAT	CCTTGGAATGTCTTTCTCCACAG

### Quantification of RELMα and type 2 cytokines

The levels of RELMα in the BAL were measured by sandwich ELISA, using rabbit anti-mouse RELMα and biotinylated rabbit anti-mouse RELMα (Peprotech). Cytokines IL-5 and IL-13 were measured in single-cell suspensions of splenocytes stimulated with *N*. *brasiliensis* excretory secretory antigen or anti-CD3 mitogen for 72hrs. IL-4 levels were measured using rat anti-mouse IL-4 (11B11, Bio X Cell) and biotinylated rat anti-mouse IL-4 (BVD6-24G2, Biolegend) compared to a recombinant IL-4 standard (Peprotech). IL-5 levels were measured using rat anti-mouse IL-5 (TRFK4, home-grown) and biotinylated rat anti-mouse IL-5 (Biolegend) compared to recombinant IL-5 (Peprotech). IL-13 levels were measured using rat anti-mouse IL-13 and biotinylated rat anti-mouse IL-13 (eBioscience) compared to a recombinant IL-13 standard (Peprotech).

### Flow cytometry

A fragment of the right lung lobe was digested for 30 min at 37°C with 0.2U/mL Liberase TL (Roche) and 80U/mL DNase (Life Tech) in Hanks Balanced Salt Solution (Sigma) prior to forcing the tissue suspensions through gauze. Red blood cells were lysed and live cells counted using trypan blue exclusion on an automated Cellometer T4 (Nexcelom). Cells were incubated with Fc block (CD16/CD32 (eBioscience) and mouse serum) and were stained with fluorescence-conjugated antibodies. Cells were identified by expression of surface markers as follows and as indicated in (S1b Fig): neutrophils Ly6G+ (1A8) CD11b+ (M1/70), dendritic cells CD11c^+^ (N418) MHCII^+^ (M5/114.15.2) F4/80^-^ (BM8), monocyte derived DCs F4/80^+^ CD11b^+^ CD11c^+^ SigF^-^ (E50-2440) alveolar macrophages F4/80^+^ CD11c^+^ CD11b^lo^, SigF^+^, interstitial macrophages F4/80^+^ CD11b^+^ CD11c^-^ SigF^-^, CD4 T cells CD4^+^ (GK1.5) TCRβ^+^ (H57-597) CD11b^-^ and ILC2s Lineage^-^ (CD11b, TCRβ TCRγδ (GL3) Ly6G F4/80 CD11c SigF CD19 (6D5)) CD90.2^+^ (30-H12) ICOS^+^ (C398.4A). Cells were fixed for 10min (RT) with 2% paraformaldehyde and stored at 4°C until intracellular staining was performed or cells were acquired.

To measure intracellular Ym1 and RELMα, cells were permeabilised (eBioscience) and incubated with rabbit anti-mouse RELMα (Peprotech) or biotinylated goat anti-mouse Ym1 (R&D) followed by Alexa-Fluor 488 rabbit xenon labeling kit (Life Technologies) and streptavidin PerCP (Biolegend). Intracellular IL-5 and IL-13 were measured in cells simulated at 37°C for 4h with PMA (phorbol myristate acetate; 0.5μg/mL) and ionomycin (1μg/mL) and for the last 3h with brefeldin A (10μg/mL). Cell surfaces were stained according to details above, fixed with 2% paraformaldehyde prior to cell permeabilisation (eBioscience). Cells were then stained with Pe-Cy7 conjugated anti-mouse IL-13 (eBio13A; eBioscience) and APC conjugated anti-mouse IL-5 (TRFK5; Biolegend) or isotype matched controls (eBRG1; eBioscience) prior to acquisition. Live/dead aqua (Life Technologies) was used to exclude dead cells from the analysis. Samples were acquired with a FACSCanto II or LSR II (Becton-Dickinson) and analysed using FlowJo software (version 9.9.5; TreeStar Inc.).

### Histology and immunofluorescence

Lung tissue was fixed-perfused with 10% neutral buffered formalin and incubated overnight prior to placing tissue in 70% ethanol. Lung tissue was processed, embedded in paraffin, and sectioned to slides. Sections were stained with hematoxylin and eosin and linear means intercept (Lmi) quantified as a score of lung damage, as described previously [9]. Briefly, lung samples were viewed by microcopy with an original magnification of ×200; 15 random non-overlapping fields per sample were assessed. Six horizontal lines were drawn across each image with ImageJ (version 1.44) and the total number of times the alveolar wall intercepted per line was counted. Line length was then divided by the number of intercepts to calculate Lmi. All samples were analyzed by researchers ‘blinded’ to sample identity. Hemosiderin Laden macrophages were assessed in sections stained with Prussian blue according to standard laboratory procedures. The numbers of Prussian blue positive macrophages were counted (x200 magnification) by a researcher “blinded” to sample identity. For immunofluorescence imaging, sections were deparaffinized, hydrated and incubated with Retrievagen A pH6.0 solution (BD Bioscience) for 20min at 98°C for antigen retrieval. Endogenous biotin was blocked (Life Technologies) prior to an overnight incubation with primary antibodies rabbit anti-mouse RELMα (1:100) and biotinylated goat anti-mouse Ym1 (1:50) or goat anti-LH2b (1:100, Santa Cruz sc-50067) followed by a 1hr incubation with Northern Lights 494 (1:100) and streptavidin NL557 (1:800), or Northern Lights 557 anti-goat (1:100). Sections were mounted with Fluormount G containing DAPI, for DNA staining. RELMa and Ym1 staining was visualised on a Leica SP5 confocal laser scanning microscope or EVOS^™^ FL Imaging System (ThermoFisher Scientific). For quantification of RELMα fluorescence intensity, three airways of similar size per sample were selected by visualisation of DNA (DAPI) by an investigator blind to sample identity. Fluorescence intensity was calculated with ImageJ software (version 1.44), by setting a threshold measurement to calculate integrated density and area of RELMα positivity corrected for background intensity.

### Statistics

Statistical analysis was performed using Prism 7.0 (version 7.0c, GraphPad Software). Differences between groups were determined by t-test or ANOVA followed by Tukey’s or Sidak multiple comparison-test. In some cases data was log-transformed to achieve normal distribution as determined by optical examination of residuals. Comparisons of different Ym1 and RELMα positive cell populations within the lungs of one experimental animal were considered as paired observations. Differences were assumed statistically significant for *P* values less than 0.05.

## Supporting information

S1 Fig IL-4Rα-dependence of Ym1 and RELMα expression in the lungs. (**a**) Microscopy of lung parenchyma sections from WT and *Il4ra*-/- BALB/c mice infected with *N*. *brasiliensis* at day 4 and 6, stained with Ym1, red; and RELMα, green (scale bars, 70μm). (**b**) flow cytometry gating strategy to identify different cell populations in the lung. Representative FACs plots from BALB/c wild-type *N*. *brasiliensis* infected mouse. (**c**) Total numbers of live single myeloid lung populations expressing intracellular Ym1 or RELMα from WT and *Il4ra*-/- uninfected (UI) mice or mice infected with *N*. *brasiliensis* (day 6); (*n* = 6 per group; data are shown as mean ± sem; two-way ANOVA with Tukey multi-comparison test; *P<0.05, **P<0.01, ***P<0.001 compared to UI wild-type (WT); #P<0.05, ## P<0.01 ###P<0.001 infected wild-type compared to infected *Il4ra* -/- mice; data are representative of 2 independent experiments, cell numbers are normalized to lung weight); AMs, alveolar macrophage; IMs, interstitial macrophage; MoDCs, monocyte derived dendritic cells; DCs, dendritic cells; Neuts, neutrophils; Eos, eosinophils. (**d**) Pie chart showing the percentage contribution of different Ym1 and RELMα^+^ cell populations in lung myeloid cells from mice as in **c**.
(TIF)

S2 Fig Ym1 alters type 2 cytokine secretion by splenocytes but adaptive Ym1 has no effect on gut parasite burden. **(a)** IL-4, IL-5 and IL-13 levels in supernatants of splenocytes from mice uninfected (UI) or *N*. *brasiliensis* infected mice treated with IgG2a isotype or anti-Ym1 at days -1 to 2 and collected at day 6. Splenocytes were cultured with medium, *N*. *brasiliensis* excretory secretory antigen (antigen; 1μg/mL) or anti-CD3 (1μg/mL) (*n* = 6 per group; data are shown as mean ± sem; two-way ANOVA with Tukey multi-comparison test; NS not significant, **P<0.01; data are representative of 2 independent experiments). (**b**) Pie chart showing the proportion of type 2 cytokine expressing CD4 T cells or ILCs in the lungs of mice uninfected (UI) or *N*. *brasiliensis* infected mice treated with IgG2a isotype or anti-Ym1 treatment at days 3 to 5 and collected at day 6 post-infection. (**c**) IL-4, IL-5 and IL-13 levels in supernatants of splenocytes from mice as in **b**. Splenocytes were cultured the same way as stated for **a** (*n* = 6 per group; data are shown as mean ± sem; two-way ANOVA with Tukey multi-comparison test; NS not significant, *P<0.05; data are representative of 2 independent experiments). **(d)** Numbers of *N*. *brasiliensis* parasites at day 6 in the small intestine of mice treated with IgG2a isotype or anti-Ym1 days 3 to 5 (*n* = 12 per group; data are shown as mean ± sem; data are pooled from 2 independent experiments). (**e**) Total Ym1 amounts in the BAL in Balb/c WT mice uninfected (UI) or N. brasiliensis infected mice at day 6 (n = 12–15 mice per group; unpaired t-test, **** P<0.0001; data pooled from 2 independent experiments). (**f**) Numbers of *N*. *brasiliensis* parasites at day 6 in the small intestine of wild-type of *Il4ra*-/- mice treated with PBS or rYm1 (8μg) intranasally on days 4 and 5 post-infection (*n* = 5–6 animals per group; data are shown as mean ± sem; data are representative of 2 independent experiments).
(TIF)

S3 Fig Impact of altered RELMα levels on lung repair in *Retnla*^+/-^ mice. **(a)** Serum levels of RELMα in naive *Retnla* +/+ or *Retnla* +/- mice (data points represent individual mice and lines show mean ± sem). (**b**) Wild-type (WT) and Retnla+/- mice uninfected or infected with *N*. *brasiliensis* (250 L3’s) and lung repair assessed at days 6 and 10. Quantification of lung damage was calculated as linear means intercept from H&E stained lung sections and values normalised to Lmi in uninfected wild-type mice (*n* = 4–8 per group; data are shown as mean ± sem; two-way ANOVA with Sidak multi-comparison test; *P<0.05 and ***P<0.001 compared to WT uninfected mice and #P<0.05 and ##P<0.01 compared to WT infected mice at each time point). Wild-type mice were a mix of *Retnla*^+/+^ and C57BL6/J mice, no statistical difference in Lmi was observed between these two strains. (c) Representative microscopy of lung sections from WT and *Retnla*^+/-^ mice as in **b**, and stained with hematoxylin and eosin. (images are representative of *n* = 4–8; scale bars, 200μm).
(TIF)

S4 Fig RELMα expression in lungs from heterozygote mice. **(a)** Expression of *Retnla* mRNA in whole lung tissue of uninfected (UI) or *N*. *brasiliensis* (500 L3) infected *Retnla*+/+ *Retnla*+/- and *Retnla*-/- mice at day 6 post-infection (*n* = 5–8 per group; data are shown as mean ± sem; one-way ANOVA with Sidak multi-comparison test; *P<0.05, **P<0.01 ****P<0.0001 compared to UI *Retnla*+/+; #P<0.05, ####P<0.0001 compared to infected *Retnla*+/+ mice; data representative of 2 independent experiments). (**b**) Frequency of RELMα+ myeloid cells in lung tissue from mice as in **a**, analysed by intracellular flow cytometry (*n* = 5–8 per group; data are shown as mean ± sem; level of RELMα positivity was set from cells stained with rabbit IgG isotype; MoDCs, monocyte-derived dendritic cells; DCs, dendritic cells; Neuts, neutrophils. (**c**) Microscope images of lung sections from infected mice as in **a** stained with the DNA-binding dye (DAPI), blue and RELMα, green. (Images are representative of 5–8 individual mice per group).
(TIF)

S5 Fig RELMα alters type 2 cytokine production by CD4+ T cells in the lung, but only at day 10 post-infection. The number of (**a & b**) ILC2s or **(c)** CD4+ T cells expressing intracellular IL-5 or IL-13 within the lungs of *Retnla* littermate mice uninfected (UI) or infected with *N*. *brasiliensis* (500 L3’s) at **(a)** 4 or **(b & c)** 6 days post infection. Single cell lung suspensions were stimulated ex vivo with PMA and ionomycin. graphs show absolute number of cytokine positive cells per g of lung tissue (*n* = 6–8 per group; data are shown as mean ± sem and are representative of 2 independent experiments). (**d**) The number of IL-13, IL-5 and IL-4 –positive CD4+ T cells in the lungs of wild-type or Retnla+/- mice uninfected (UI) or infected with *N*. *brasiliensis* (250 L3’s) at day 6 or day 10 post-infection. (*n* = 4–8 per group; data are shown as mean ± sem; one-way ANOVA with Sidak multi-comparison test; NS not significant and *P<0.05.
(TIF)

S6 Fig RELMα-deficiency enhances type 2 cytokines production by restimulated splenocytes. **(a)** IL-4, **(b)** IL-5 and **(c)** IL-13 levels in supernatants of splenocytes from mice uninfected (UI) or *N*. *brasiliensis* infected *Retnla* littermate mice collected at day 4 or 6. Splenocytes were cultured with medium, *N*. *brasiliensis* excretory secretory antigen (antigen; 1μg/mL) or anti-CD3 (1μg/mL) (*n* = 6 per group; data are shown as mean ± sem; two-way ANOVA with Tukey multi-comparison test; NS not significant, *P<0.05 and **P<0.01 ***P<0.001; data are representative of 2 independent experiments).
(TIF)

S7 Fig Proposed mechanisms by which Ym1 and RELMα regulate immune responses and tissue pathology. During early time points following infection with *N*. *brasiliensis* (day 2), innate Ym1 expression, predominantly via alveolar macrophages (AMs) and neutrophils (Neuts) promotes IL-17 production from innate γδT cells and subsequent neutrophilic recruitment into the lungs [9]. Whilst neutrophils, together with larval migration, cause lung damage, IL-17 promotes type 2 cytokine expression from both innate lymphoid cells (ILCs) and CD4+ T cells. Thereby, innate Ym1 enhances type 2 responses that rapidly contribute to resolving tissue damage. Once *N*. *brasiliensis* larvae have passed through the lung tissue, a strongly polarised type 2 response occurs and enhanced expression of Ym1 is evident not only from AMs and Neuts, but also dendritic cells (DCs), interstitial macrophages (IMs) and monocyte-derived dendritic cells (MoDCs). During this adaptive lung environment, Ym1 drives epithelial-derived RELMα which promotes lung repair via regulation of collagen fibril formation. It is likely that Ym1 also regulates tissue repair via other mechanisms associated with its ability to bind extracellular matrix. To ensure excessive type 2 cytokine production does not become pathogenic and induce fibrotic responses, Ym1 either via induction of RELMα or other mechanisms, negatively regulates type 2 cytokines levels.
(TIF)
