# Supplementary figures and images for: Ym1 induces RELMα and rescues IL-4Rα deficiency in lung repair during nematode infection
Source: PLoS Pathog. 2018 Nov 30;14(11):e1007423. doi: 10.1371/journal.ppat.1007423 (PMC6291165; doi:10.1371/journal.ppat.1007423)

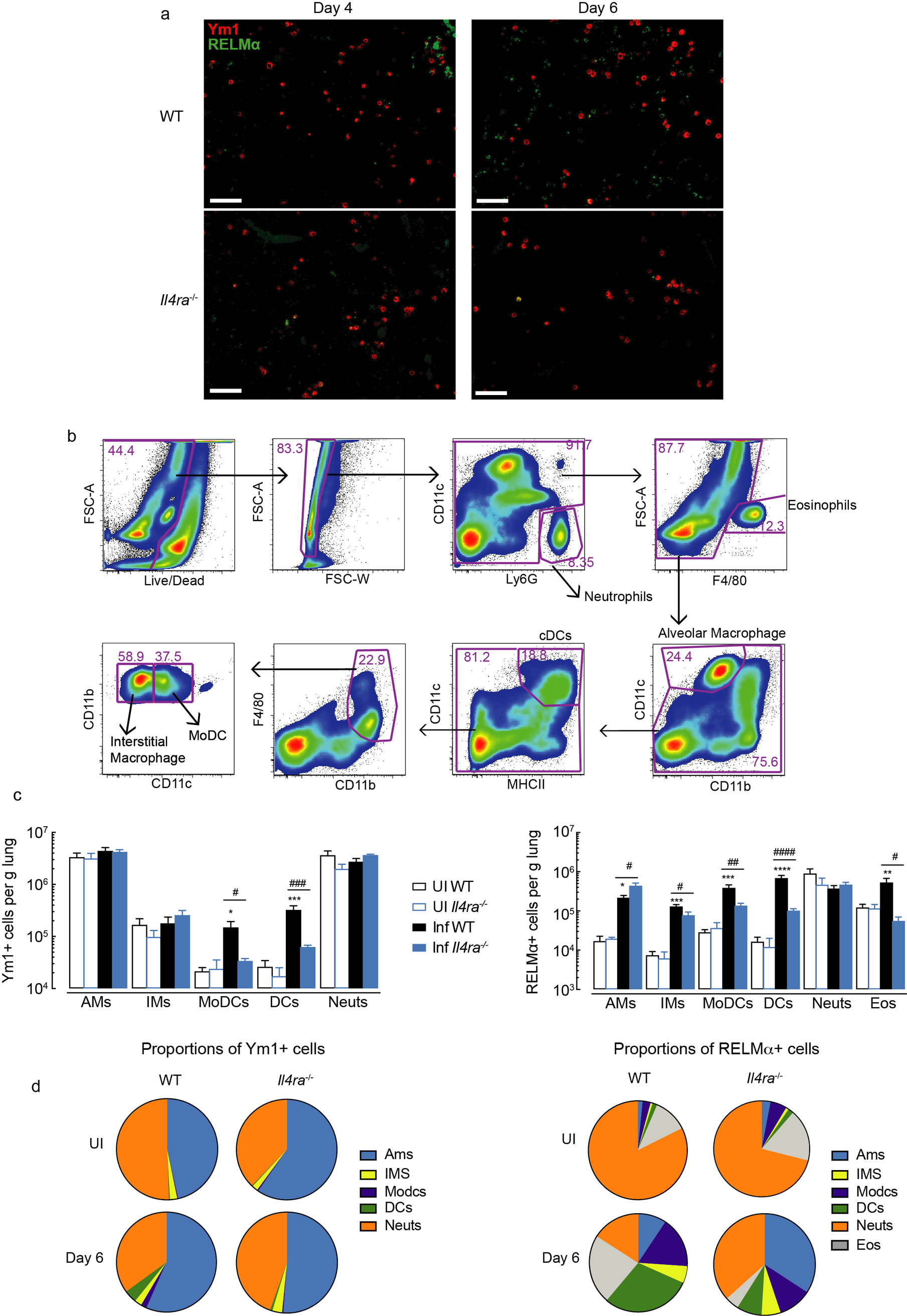

Supplement: S1 Fig — (a) Microscopy of lung parenchyma sections from WT and Il4ra-/- BALB/c mice infected with N. brasiliensis at day 4 and 6, stained with Ym1, red; and RELMα, green (scale bars, 70μm). (b) flow cytometry gating strategy to identify different cell populations in the lung. Representative FACs plots from BALB/c wild-type N. brasiliensis infected mouse. (c) Total numbers of live single myeloid lung populations expressing intracellular Ym1 or RELMα from WT and Il4ra-/- uninfected (UI) mice or mice infected with N. brasiliensis (day 6); (n = 6 per group; data are shown as mean ± sem; two-way ANOVA with Tukey multi-comparison test; *P<0.05, **P<0.01, ***P<0.001 compared to UI wild-type (WT); #P<0.05, ## P<0.01 ###P<0.001 infected wild-type compared to infected Il4ra -/- mice; data are representative of 2 independent experiments, cell numbers are normalized to lung weight); AMs, alveolar macrophage; IMs, interstitial macrophage; MoDCs, monocyte derived dendritic cells; DCs, dendritic cells; Neuts, neutrophils; Eos, eosinophils. (d) Pie chart showing the percentage contribution of different Ym1 and RELMα+ cell populations in lung myeloid cells from mice as in c. (TIF) [file ppat.1007423.s001.tif]

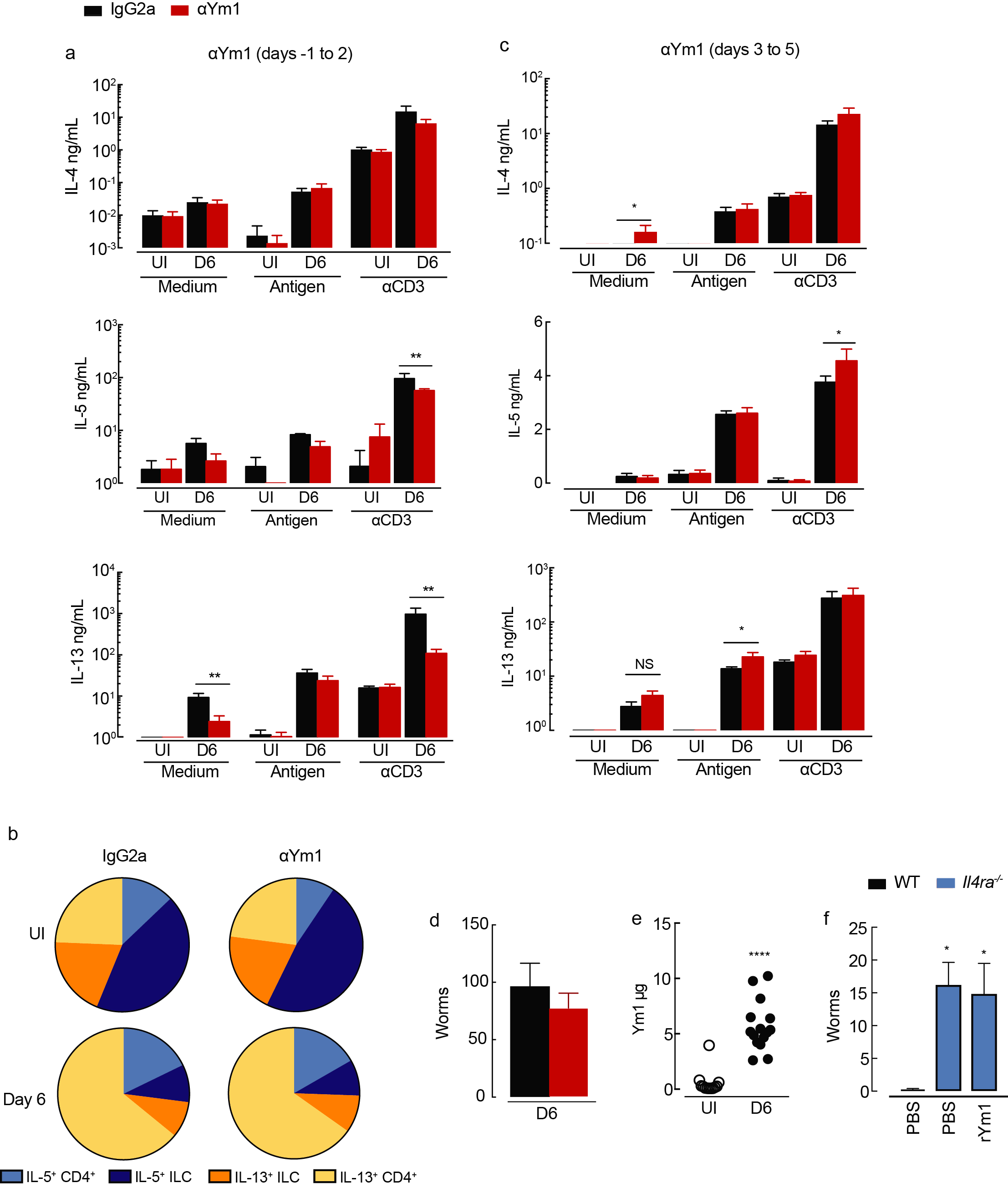

Supplement: S2 Fig — (a) IL-4, IL-5 and IL-13 levels in supernatants of splenocytes from mice uninfected (UI) or N. brasiliensis infected mice treated with IgG2a isotype or anti-Ym1 at days -1 to 2 and collected at day 6. Splenocytes were cultured with medium, N. brasiliensis excretory secretory antigen (antigen; 1μg/mL) or anti-CD3 (1μg/mL) (n = 6 per group; data are shown as mean ± sem; two-way ANOVA with Tukey multi-comparison test; NS not significant, **P<0.01; data are representative of 2 independent experiments). (b) Pie chart showing the proportion of type 2 cytokine expressing CD4 T cells or ILCs in the lungs of mice uninfected (UI) or N. brasiliensis infected mice treated with IgG2a isotype or anti-Ym1 treatment at days 3 to 5 and collected at day 6 post-infection. (c) IL-4, IL-5 and IL-13 levels in supernatants of splenocytes from mice as in b. Splenocytes were cultured the same way as stated for a (n = 6 per group; data are shown as mean ± sem; two-way ANOVA with Tukey multi-comparison test; NS not significant, *P<0.05; data are representative of 2 independent experiments). (d) Numbers of N. brasiliensis parasites at day 6 in the small intestine of mice treated with IgG2a isotype or anti-Ym1 days 3 to 5 (n = 12 per group; data are shown as mean ± sem; data are pooled from 2 independent experiments). (e) Total Ym1 amounts in the BAL in Balb/c WT mice uninfected (UI) or N. brasiliensis infected mice at day 6 (n = 12–15 mice per group; unpaired t-test, **** P<0.0001; data pooled from 2 independent experiments). (f) Numbers of N. brasiliensis parasites at day 6 in the small intestine of wild-type of Il4ra-/- mice treated with PBS or rYm1 (8μg) intranasally on days 4 and 5 post-infection (n = 5–6 animals per group; data are shown as mean ± sem; data are representative of 2 independent experiments). (TIF) [file ppat.1007423.s002.tif]

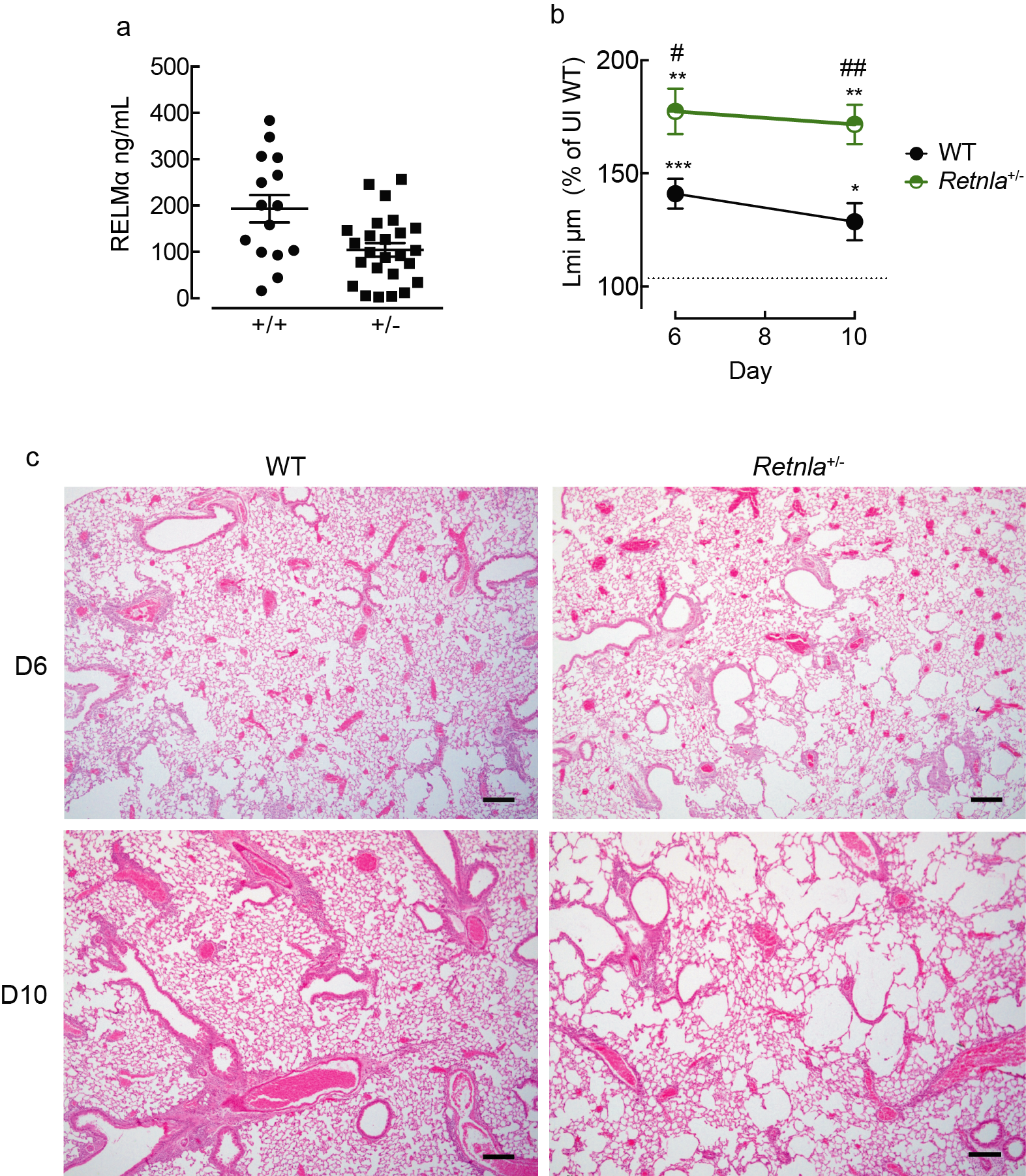

Supplement: S3 Fig — (a) Serum levels of RELMα in naive Retnla +/+ or Retnla +/- mice (data points represent individual mice and lines show mean ± sem). (b) Wild-type (WT) and Retnla+/- mice uninfected or infected with N. brasiliensis (250 L3’s) and lung repair assessed at days 6 and 10. Quantification of lung damage was calculated as linear means intercept from H&E stained lung sections and values normalised to Lmi in uninfected wild-type mice (n = 4–8 per group; data are shown as mean ± sem; two-way ANOVA with Sidak multi-comparison test; *P<0.05 and ***P<0.001 compared to WT uninfected mice and #P<0.05 and ##P<0.01 compared to WT infected mice at each time point). Wild-type mice were a mix of Retnla+/+ and C57BL6/J mice, no statistical difference in Lmi was observed between these two strains. (c) Representative microscopy of lung sections from WT and Retnla+/- mice as in b, and stained with hematoxylin and eosin. (images are representative of n = 4–8; scale bars, 200μm). (TIF) [file ppat.1007423.s003.tif]

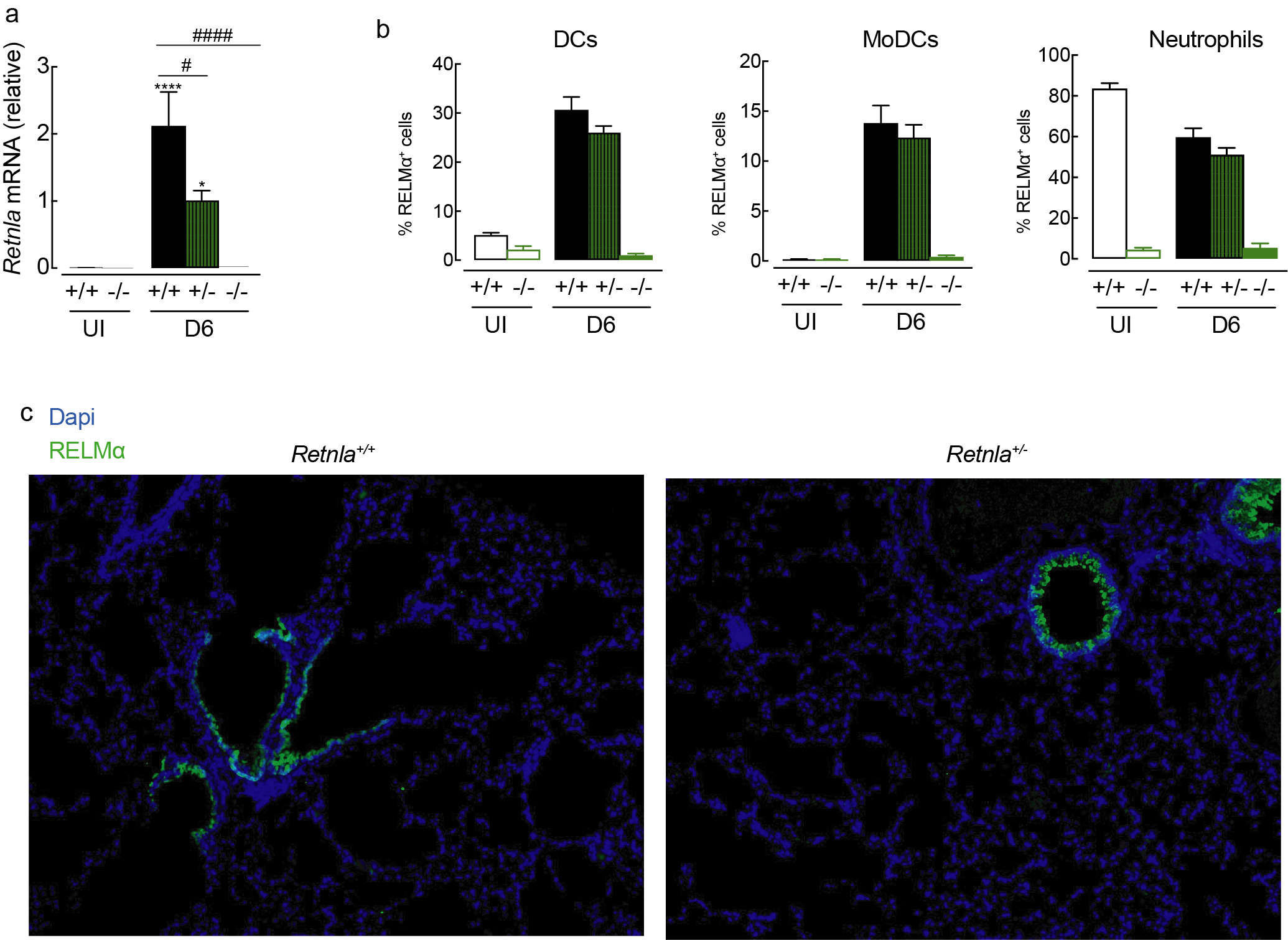

Supplement: S4 Fig — (a) Expression of Retnla mRNA in whole lung tissue of uninfected (UI) or N. brasiliensis (500 L3) infected Retnla+/+ Retnla+/- and Retnla-/- mice at day 6 post-infection (n = 5–8 per group; data are shown as mean ± sem; one-way ANOVA with Sidak multi-comparison test; *P<0.05, **P<0.01 ****P<0.0001 compared to UI Retnla+/+; #P<0.05, ####P<0.0001 compared to infected Retnla+/+ mice; data representative of 2 independent experiments). (b) Frequency of RELMα+ myeloid cells in lung tissue from mice as in a, analysed by intracellular flow cytometry (n = 5–8 per group; data are shown as mean ± sem; level of RELMα positivity was set from cells stained with rabbit IgG isotype; MoDCs, monocyte-derived dendritic cells; DCs, dendritic cells; Neuts, neutrophils. (c) Microscope images of lung sections from infected mice as in a stained with the DNA-binding dye (DAPI), blue and RELMα, green. (Images are representative of 5–8 individual mice per group). (TIF) [file ppat.1007423.s004.tif]

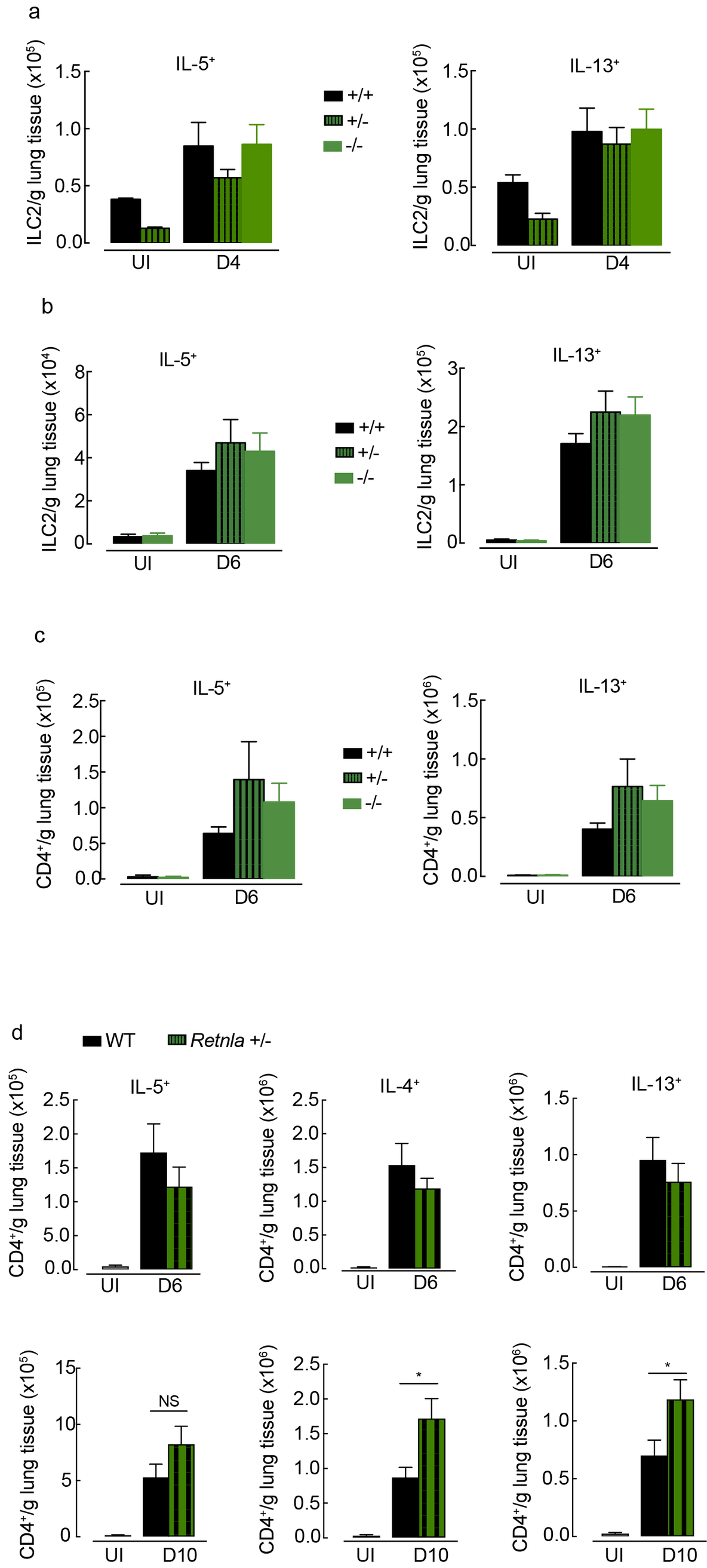

Supplement: S5 Fig — The number of (a & b) ILC2s or (c) CD4+ T cells expressing intracellular IL-5 or IL-13 within the lungs of Retnla littermate mice uninfected (UI) or infected with N. brasiliensis (500 L3’s) at (a) 4 or (b & c) 6 days post infection. Single cell lung suspensions were stimulated ex vivo with PMA and ionomycin. graphs show absolute number of cytokine positive cells per g of lung tissue (n = 6–8 per group; data are shown as mean ± sem and are representative of 2 independent experiments). (d) The number of IL-13, IL-5 and IL-4 –positive CD4+ T cells in the lungs of wild-type or Retnla+/- mice uninfected (UI) or infected with N. brasiliensis (250 L3’s) at day 6 or day 10 post-infection. (n = 4–8 per group; data are shown as mean ± sem; one-way ANOVA with Sidak multi-comparison test; NS not significant and *P<0.05. (TIF) [file ppat.1007423.s005.tif]

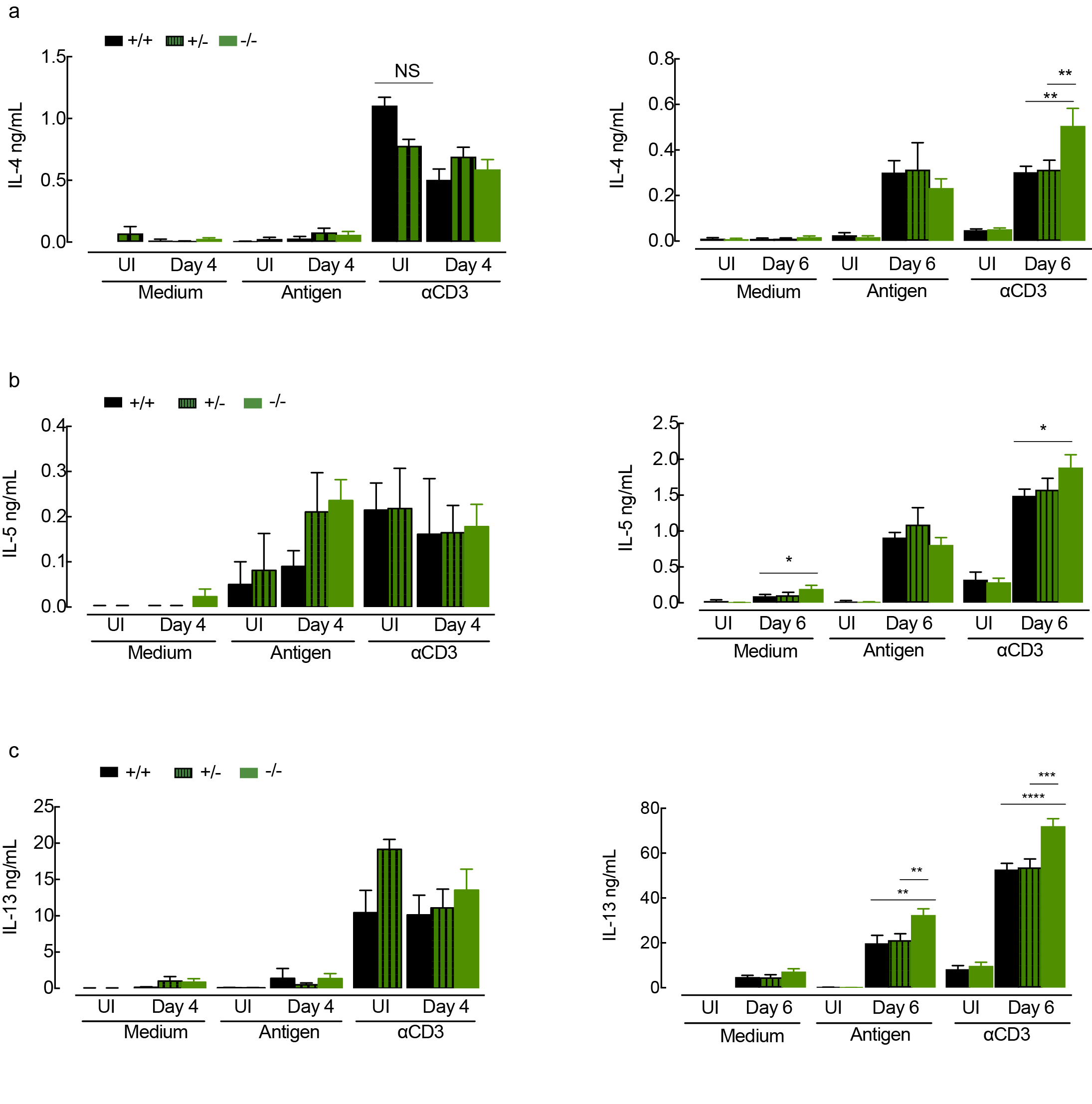

Supplement: S6 Fig — (a) IL-4, (b) IL-5 and (c) IL-13 levels in supernatants of splenocytes from mice uninfected (UI) or N. brasiliensis infected Retnla littermate mice collected at day 4 or 6. Splenocytes were cultured with medium, N. brasiliensis excretory secretory antigen (antigen; 1μg/mL) or anti-CD3 (1μg/mL) (n = 6 per group; data are shown as mean ± sem; two-way ANOVA with Tukey multi-comparison test; NS not significant, *P<0.05 and **P<0.01 ***P<0.001; data are representative of 2 independent experiments). (TIF) [file ppat.1007423.s006.tif]

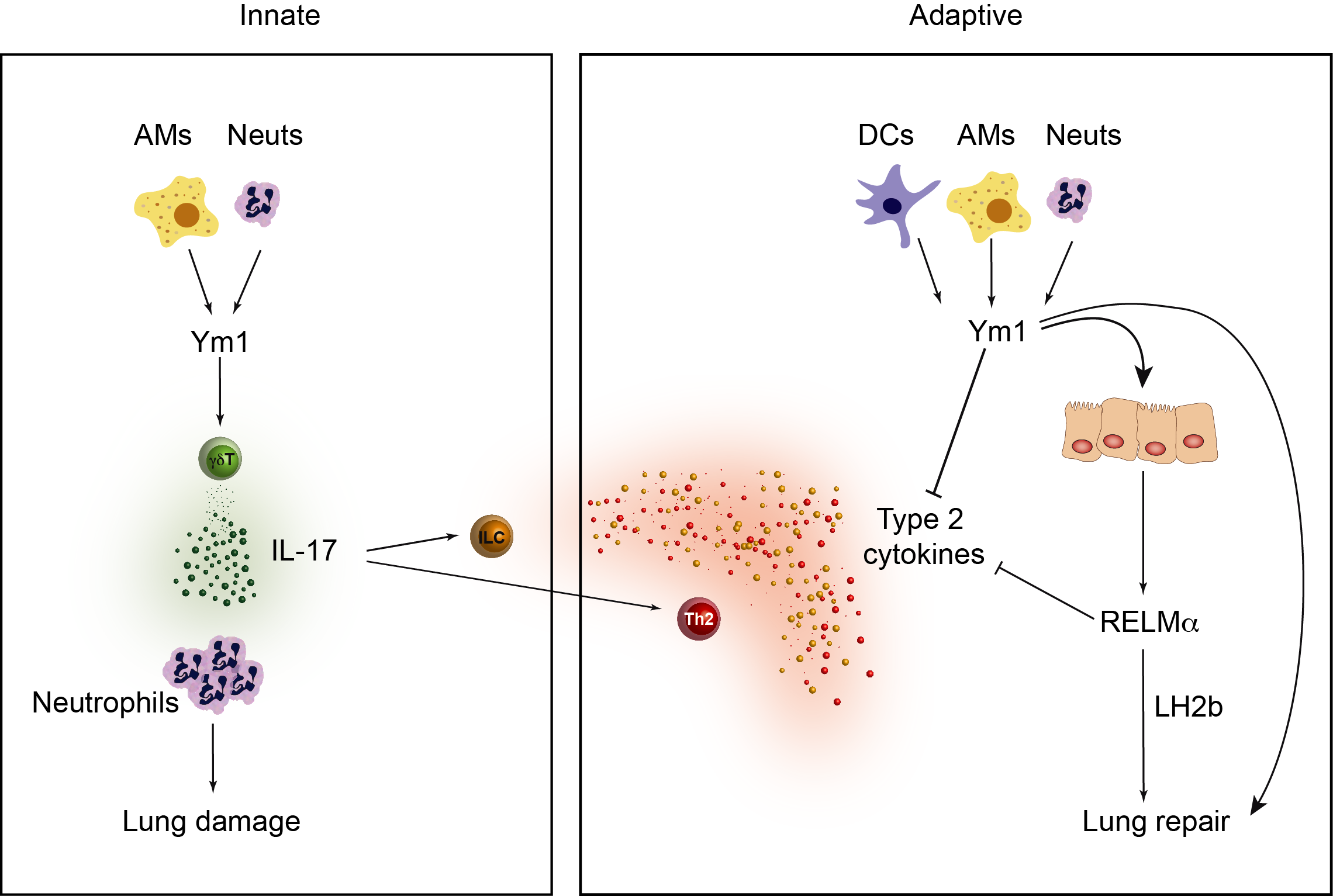

Supplement: S7 Fig — During early time points following infection with N. brasiliensis (day 2), innate Ym1 expression, predominantly via alveolar macrophages (AMs) and neutrophils (Neuts) promotes IL-17 production from innate γδT cells and subsequent neutrophilic recruitment into the lungs [9]. Whilst neutrophils, together with larval migration, cause lung damage, IL-17 promotes type 2 cytokine expression from both innate lymphoid cells (ILCs) and CD4+ T cells. Thereby, innate Ym1 enhances type 2 responses that rapidly contribute to resolving tissue damage. Once N. brasiliensis larvae have passed through the lung tissue, a strongly polarised type 2 response occurs and enhanced expression of Ym1 is evident not only from AMs and Neuts, but also dendritic cells (DCs), interstitial macrophages (IMs) and monocyte-derived dendritic cells (MoDCs). During this adaptive lung environment, Ym1 drives epithelial-derived RELMα which promotes lung repair via regulation of collagen fibril formation. It is likely that Ym1 also regulates tissue repair via other mechanisms associated with its ability to bind extracellular matrix. To ensure excessive type 2 cytokine production does not become pathogenic and induce fibrotic responses, Ym1 either via induction of RELMα or other mechanisms, negatively regulates type 2 cytokines levels. (TIF) [file ppat.1007423.s007.tif]
